# A comprehensive review of emerging therapeutic strategies against methicillin-resistant *Staphylococcus aureus*

**DOI:** 10.3389/fmicb.2026.1815573

**Published:** 2026-08-04

**Authors:** V. Keerthi, Paripoorani Ravindran, Shreeja Kaliyur, Sri Uma Sneha Tuttagunta, Shalini Mathpal, Tushar Joshi, Sudha Ramaiah, Anand Anbarasu

**Affiliations:** 1 Department of Biotechnology, School of Biosciences and Technology, Vellore Institute of Technology (VIT), Vellore, Tamil Nadu, India; 2 Department of Bio-Sciences, School of Biosciences and Technology, Vellore Institute of Technology (VIT), Vellore, Tamil Nadu, India

**Keywords:** biomimetic nano-NETs, CRISPR-Cas, *mecA* gene, methicillin-resistant *Staphylococcus aureus*, PBP2a

## Abstract

Methicillin-resistant *Staphylococcus aureus* (MRSA) remains one of the most significant multidrug-resistant bacterial pathogens responsible for a broad spectrum of infections ranging from mild skin infections to severe invasive diseases, including bacteremia, pneumonia, endocarditis, osteomyelitis, and sepsis. The rapid global dissemination of MRSA is primarily driven by the acquisition of the *mecA* gene encoding penicillin-binding protein 2a, which confers resistance to *β*-lactam antibiotics. In addition to β-lactam resistance, MRSA exhibits resistance to multiple antimicrobial classes through diverse mechanisms, including target-site mutations, efflux pumps, biofilm formation, horizontal gene transfer, and adaptive phenotypic variations. The virulence and persistence of MRSA is further enhanced by numerous virulence factors such as adhesins, toxins, immune evasion proteins, and extracellular enzymes that facilitate colonization, persistence, and host tissue damage. Biofilm formation additionally contributes to chronic infection and antibiotic tolerance. Despite the availability of conventional agents such as vancomycin, linezolid, and daptomycin, the emergence of resistant strains including vancomycin-resistant *Staphylococcus aureus* has significantly limited current therapeutic options. Consequently, there is an urgent need for innovative therapeutic strategies. This review comprehensively summarizes the evolution, pathogenesis, virulence mechanisms, biofilm biology, and antibiotic resistance mechanisms of MRSA, with particular emphasis on emerging therapeutic approaches. Novel strategies including antimicrobial peptides, nanomedicine, bacteriophage therapy, CRISPR-Cas systems, biomimetic nano-NETs, probiotics, monoclonal antibodies and plant-derived compounds are discussed as promising alternatives or adjuncts to conventional antibiotics. Collectively, these advances highlight the evolving landscape of MRSA management and the potential for next-generation therapeutics to overcome antimicrobial resistance challenges.

## 1 Introduction

*Staphylococcus aureus* (*S. aureus*) is a Gram-positive opportunistic pathogen capable of causing a wide range of infections, from mild skin and soft tissue infections to severe invasive diseases such as bacteremia, endocarditis, pneumonia, osteomyelitis, and toxic shock syndrome (Deurenberg and Stobberingh, 2008; D’Souza et al., 2010). Although *S. aureus* is a commensal organism on the skin and in the nasal passages, it demonstrates its adaptability and persistence by taking advantage of wounds in host barriers or immune defenses to create invasive infections (Laux et al., 2019). Initially, *S. aureus* infections were treated with penicillin (Chambers and Deleo, 2009). However, widespread use led to the rapid emergence of resistant strains, promoting the development of methicillin as an alternative *β*-lactam antibiotic. Subsequently, methicillin-resistant *Staphylococcus aureus* (MRSA) emerged, exhibiting resistance to most β-lactam antibiotics. MRSA was first reported in 1961, just 2 years after methicillin was introduced into clinical practice (Barber, 1961). Resistance in MRSA is primarily mediated by the *mecA* gene, which is carried on a mobile genetic element known as the staphylococcal cassette chromosome (SCCmec). The *mecA* gene encodes penicillin-binding protein 2a (PBP2a), a modified PBP with reduced affinity for β-lactam antibiotics, thereby rendering most antibiotics in this class ineffective (Lade and Kim, 2023). This mechanism has contributed to the widespread dissemination of MRSA globally.

MRSA is one of the most prevalent antibiotic-resistant pathogens worldwide, with high incidence reported across regions including Asia, the Middle East, Europe, and the Americas (Bashabsheh et al., 2024). MRSA has been classified as a high-priority threat and is associated with approximately 100,000 deaths annually (European Antimicrobial Resistance Collaborators, 2022). According to the Centers for Disease Control and Prevention (CDC), MRSA remains a serious threat to public health, which is responsible for more than 70,000 severe infections and approximately 9,000 deaths annually in the United States. Recent surveillance data also indicates that during 2023, hospital-onset MRSA infections in acute care hospitals declined by 16% (Centers for Disease Control and Prevention, 2025). Furthermore, according to the WHO’s Global Antimicrobial Resistance and Use Surveillance System (GLASS) report, the global prevalence of MRSA bloodstream infections in 2023 was 27.1% (23.5–31.0). The highest prevalence was recorded in the Eastern Mediterranean region at 50.3% (39.8–60.8), highlighting the substantial global burden of MRSA. In the decades following its initial emergence, MRSA established itself as a major nosocomial pathogen, particularly within healthcare settings (WHO Antimicrobial Resistance Division, 2025). Based on epidemiological and molecular characteristics, MRSA strains are broadly classified into hospital-acquired (HA-MRSA), community-acquired (CA-MRSA), and livestock-associated (LA-MRSA). HA-MRSA is typically associated with healthcare settings and multidrug resistance, whereas CA-MRSA affects otherwise healthy individuals and often carries virulence factors such as Panton–Valentine leukocidin (PVL). PVL is a cytotoxin that is associated with increased severity of skin and soft tissue infections. LA-MRSA has emerged in individuals with close animal contact, highlighting its zoonotic potential (Takizawa et al., 2005; Shoaib et al., 2022).

The treatment of MRSA infections relies on a limited number of antibiotics. Vancomycin has historically served as the primary therapeutic agent due to its ability to inhibit Gram-positive cell wall biosynthesis (Larsen et al., 2022). However, the emergence of vancomycin-resistant *Staphylococcus aureus* (VRSA) has significantly compromised its clinical effectiveness and led to treatment failures (Chen et al., 2013). Alternative agents such as daptomycin and linezolid have been introduced to manage resistant infections, along with topical agents like mupirocin and newer antibiotics including tigecycline, dalbavancin, oritavancin, and ceftaroline (Toh et al., 2007; Mendes et al., 2008; Locke et al., 2014).

Despite these therapeutic advancements, the effectiveness of currently available antibiotics has declined substantially, posing significant challenges in MRSA treatment and infection management (Vuong et al., 2016). MRSA frequently exhibits resistance not only to β-lactam antibiotics but also to other antimicrobial classes such as macrolides, aminoglycosides, and fluoroquinolones, further limiting treatment options (Tong et al., 2015). In addition, resistance to newer agents, including linezolid, daptomycin, and ceftaroline, has also been reported, complicating clinical management (Greer, 2006; Foster, 2017). These challenges are further exacerbated by factors such as inappropriate antibiotic use, inadequate diagnostics, and environmental antibiotic exposure, particularly in resource-limited settings (DiMondi et al., 2011; Abebe and Birhanu, 2023).

These challenges have driven increasing interest in the exploration of alternative and emerging therapeutic strategies for the effective management of MRSA infections. Recent advances include next-generation antimicrobials such as tedizolid, dalbavancin, and oritavancin, along with innovative approaches such as antimicrobial peptides, bacteriophage therapy, and nanotechnology-based drug delivery systems designed to enhance therapeutic targeting and reduce the development of resistance (Kumar et al., 2025). In addition, combination therapies and immunomodulatory approaches are being investigated as adjuncts to conventional treatments to improve clinical outcomes (Hamwi and Salem-Sokhn, 2025).

This review provides a comprehensive overview of MRSA, including its epidemiology, resistance mechanisms, and clinical impact, with a particular focus on emerging therapeutic strategies. By integrating current knowledge and recent advances, this article aims to highlight the evolving landscape of MRSA management and the potential of novel approaches to address ongoing therapeutic challenges.

## 2 Evolution and emergence of MRSA

**Figure 1:**
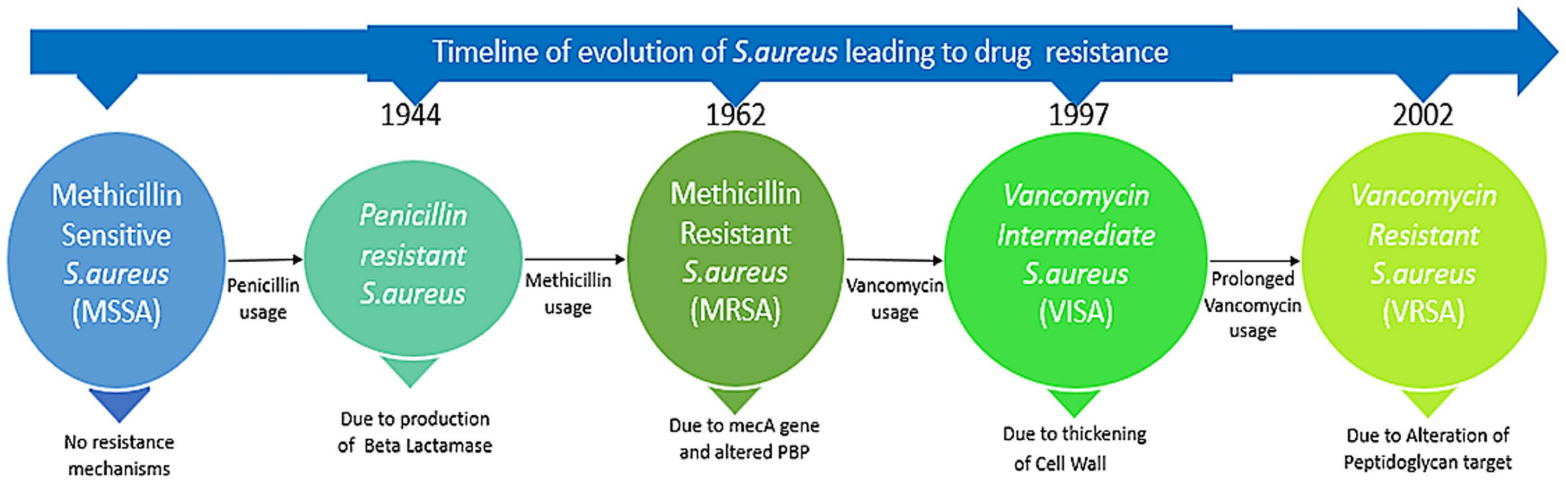
Timeline showing the evolution of antibiotic resistance in *Staphylococcus aureus* infection from methicillin-sensitive *Staphylococcus aureus* to penicillin-resistant strains, m*ethicillin-resistant Staphylococcus aureus*, vancomycin-intermediate *Staphylococcus aureus*, and vancomycin-resistant *Staphylococcus aureus*. The figure highlights the major resistance mechanisms acquired during the progressive exposure to antibiotics.

The initial clinical impact of MRSA was described in the early 1960s, shortly after methicillin was introduced into clinical practice. The first MRSA isolate was reported in the United Kingdom in 1961 (Eriksen and Erichsen, 1964). Analyses of whole-genome sequences have revealed that the genetic changes responsible for methicillin resistance, particularly the acquisition of the *mecA* gene carried on the SCCmec, occurred well before the clinical use of methicillin. Bayesian phylogenetic reconstructions indicate that the acquisition of an ancestral SCCmec element dates back to the mid-1940s. This suggests that earlier selective pressures from β-lactam antibiotics, especially the widespread use of penicillin, played a significant role in the initial development of resistance (Harkins et al., 2017). The major stages involved in the evolution of antibiotic-resistant *S. aureus infection* strains, from methicillin-susceptible *S. aureus* (MSSA) to VRSA, are illustrated in Figure 1. Following the emergence of methicillin resistance, MRSA rapidly evolved into a major nosocomial pathogen and became widely disseminated in healthcare settings worldwide.

HA-MRSA strains emerged through the clonal expansion of successful *S. aureus* lineages and spread widely across geographic regions, as demonstrated by multilocus sequence typing and population-based studies. These strains were typically multidrug-resistant and were primarily associated with healthcare-related infections, including those linked to invasive medical devices, resulting in increased morbidity among high-risk patients (Lee et al., 2018). A significant epidemiological shift occurred in the 1990s with the emergence of CA-MRSA. These strains, including the USA300 clone in North America, are capable of infecting otherwise healthy individuals without prior healthcare exposure and are commonly associated with skin and soft tissue infections as well as severe conditions such as necrotizing pneumonia. CA-MRSA strains combine methicillin resistance with enhanced virulence and fitness, which facilitates their rapid transmission within community settings. This increased virulence is often attributed to the acquisition of specific toxin genes, particularly those encoding Panton–Valentine leukocidin (PVL), along with other mobile genetic elements. The epidemiology, clinical spectrum, and transmission dynamics of CA-MRSA have been extensively described in the literature (David and Daum, 2010; Otto, 2013).

Another important stage in the evolution of MRSA was the emergence of livestock-associated MRSA (LA-MRSA) in the early 2000s, particularly clonal complex CC398 (ST398), which is commonly associated with livestock such as pigs and individuals in close contact with these animals. Genomic analyses suggest that CC398 likely originated in humans as MSSA before adapting to livestock hosts under selective pressures, including the use of antibiotics such as tetracycline, which facilitated its expansion and the acquisition of SCCmec elements. Subsequent transmission between animals and humans highlights the bidirectional spread of this lineage and underscores the importance of a One Health perspective in understanding MRSA evolution (Sharma et al., 2016). Recent genomic and ecological studies have further highlighted the complexity of MRSA evolution, with the identification of novel SCCmec variants, such as *mecC*-containing MRSA, and the recognition of wildlife reservoirs, including hedgehogs. These findings suggest that methicillin resistance in *S. aureus* may have arisen through multiple independent evolutionary events across diverse ecological niches. Notably, evidence from wildlife studies indicates that *mecC*-MRSA lineages existed prior to the antibiotic era, supporting the concept that resistance is not solely a consequence of modern antibiotic use but also reflects long-term evolutionary processes driven by both natural and anthropogenic selective pressures (Larsen et al., 2022).

Overall, the evolution of MRSA has been shaped by multiple interconnected factors, including horizontal gene transfer of SCCmec elements enabling rapid acquisition of resistance, clonal expansion of successful lineages across distinct epidemiological settings such as hospitals, communities, and livestock, and sustained selective pressures from antibiotic use and host interactions (Enright et al., 2002). These insights underscore the importance of continuous genomic surveillance, responsible antimicrobial stewardship in both human and veterinary medicine, and the adoption of a One Health approach to better understand and limit the emergence and transmission of MRSA.

## 3 Pathogenesis of *Staphylococcus aureus*

*S. aureus* is a versatile Gram-positive coccus that colonizes the skin and nasal mucosa without disease but causes a spectrum of infections when barriers are breached. *S. aureus* infection typically occurs through breaches in the skin or mucosal surfaces such as open wounds or following viral infections (e.g., influenza) that damage the respiratory epithelium, thereby rendering individuals susceptible to secondary pneumonia. Upon entering host tissues, *S. aureus* rapidly upregulates its virulence genes (Novick, 2003). Host immune cells including epithelial cells and resident phagocytes recognize bacterial components, such as peptidoglycans and lipoproteins, via pattern recognition receptors. This triggers inflammatory responses and recruits’ neutrophils and macrophages (Fournier and Philpott, 2005).

In localized infections, including skin and soft tissue infections, abscesses, wound infections, and device-associated infections, the bacterium adheres to host tissues via surface adhesins such as clumping factors and fibronectin-binding proteins, leading to intense neutrophilic inflammation and the formation of pus-filled abscesses (Thammavongsa et al., 2015). To ensure its survival, *S. aureus* employs a variety of immune evasion strategies. It avoids opsonization and phagocytosis through surface structures such as capsules, Protein A, and clumping factors as well as through the use of complement inhibitors. The bacterium can also persist intracellularly within both epithelial and immune cells (Foster, 2005; Rooijakkers et al., 2005). Neutrophils play a critical role in host defense. However, *S. aureus* counteracts them by inhibiting chemotaxis (via CHIPS), blocking adhesion (via Eap), and producing toxins that destroy immune cells. Furthermore, it resists oxidative killing through antioxidant enzymes (e.g., catalase, superoxide dismutase) and neutralizes antimicrobial peptides by altering its surface charge and secreting proteases (Chavakis et al., 2002; de Haas et al., 2004).

When local containment fails, *S. aureus* can invade the bloodstream, leading to bacteremia and systemic dissemination. In systemic infections, the bacterium survives in circulation, evades immune clearance, and spreads hematogenously to distant organs, causing severe conditions such as infective endocarditis, osteomyelitis, septic arthritis, pneumonia, and sepsis.

This transition from localized to systemic disease is facilitated by its ability to adhere to endothelial surfaces, resist host defenses, and produce toxins that promote tissue invasion and immune dysregulation. For sustained survival in both local and systemic environments, nutrient acquisition particularly iron is essential, and *S. aureus* produces siderophores and activates iron uptake systems to acquire iron under limiting conditions (Maresso and Schneewind, 2006). Despite the activation of adaptive immunity, *S. aureus* can cause recurrent infections. This is attributed to its ability to disrupt T-cell and B-cell responses through toxins such as enterotoxins, TSST, and Protein A factors that interfere with the formation of immunological memory (Llewelyn and Cohen, 2002; Goodyear and Silverman, 2004). Furthermore, biofilm formation enhances its persistence on surfaces and increases resistance to antibiotics, while small colony variants contribute to chronic and recurrent infections by surviving in a metabolically inactive state (Liu, 2009).

MRSA infection can lead to a variety of complications, such as endocarditis, necrotizing fasciitis, abscess, bacteremia, venous thrombosis, bone and joint infections, pneumonia, sepsis, and septic shock. MRSA infection is associated with numerous adverse outcomes, including prolonged ICU and hospital stays, increased disease severity, higher mortality rates, and substantial healthcare costs.

The use of Vancomycin has led to the emergence of new strains, such as hVISA, VISA, and VRSA. Given that the efficacy of antibiotics used to treat MRSA infections may be suboptimal and considering the adverse effects associated with these medications it is imperative to explore alternative treatment options (Choo and Chambers, 2016).

## 4 Virulence factors in *Staphylococcus aureus*

MRSA is a prevalent and clinically significant pathogens, whose virulence factors enable immune evasion, host colonization, and tissue damage, leading to a broad spectrum of infections. These virulence determinants primarily comprise structural components of the cell envelope, surface-associated proteins, extracellular enzymes, and secreted toxins, which collectively enhance its pathogenicity (Algammal et al., 2020). Some important surface-associated proteins and extracellular enzymes involved in pathogenicity are described below.

### 4.1 Cell envelope, surface proteins, and enzymes

The cell envelope of *S. aureus* plays a crucial role in virulence by mediating interactions with the host immune system, including complement activation, phagocytosis, and antibody-mediated opsonization. It is primarily composed of capsular polysaccharides, peptidoglycan, wall teichoic acids (WTAs), lipoteichoic acids (LTAs), and a variety of surface proteins. These components not only provide structural integrity but also contribute significantly to immune evasion and persistence within the host (Zheng et al., 2021).

Surface-associated proteins are key mediators of bacterial adhesion and colonization, facilitating attachment to host tissues and initiating infection. Important adhesins include Staphylococcal protein A (SpA), fibrinogen-binding protein (Efb), surface protein G (SasG), iron-regulated surface determinant proteins (Isd system), collagen adhesin (Cna), and fibronectin-binding proteins A and B (FnBPA and FnBPB). SpA, a surface protein with a molecular weight ranging from 40 to 60 kDa, interacts with the Fc region of mammalian IgG with varying degrees of selectivity. SpA consists of five domains, each of which can bind to both the Fc and Fab regions of IgG. This makes it even more difficult for the host’s immune system to clear MRSA colonisation (Kane et al., 2018).

Efb is a 15.6 kDa protein that acts as a virulence factor. It is secreted by *S. aureus* to evade the host’s immune system by inhibiting phagocytosis. By binding to both fibrinogen and complement C3b, these proteins can form a shield-like structure that prevents phagocytosis (Zhang et al., 2022). Cna is essential for MRSA infection, as it facilitates the bacterium’s adherence to the host and enables it to evade the immune system (Herman-Bausier et al., 2016).

The Isd system comprises four surface-anchored proteins that sequester iron: IsdA, IsdB, IsdC, and IsdH. MRSA bacteria utilize their surface hemoglobin receptors such as IsdH and IsdB to destabilize heme-binding pockets, acquire increased levels of iron, and infect the host (Valenciano-Bellido et al., 2023). FnBPA and FnBPB promote adhesion to host extracellular matrix components such as fibrinogen, elastin, histones, and fibronectin, thereby enhancing colonization and biofilm formation. These proteins are encoded by the *fnbA* and *fnbB* genes (Speziale and Pietrocola, 2020).

SasG contributes to the initial attachment of MRSA to skin corneocytes and exists in two allelic forms (SasG-I and SasG-II), with SasG-II exhibiting broader ligand-binding capacity, thereby enhancing colonization efficiency.

In addition to surface proteins, MRSA secretes several extracellular enzymes, including coagulase, hyaluronidase, nuclease, proteases, and staphylokinase, which facilitate tissue invasion and dissemination. Coagulase (Coa) and von Willebrand factor-binding protein (vWbp) activate prothrombin, leading to fibrin clot formation that shields bacteria from immune cells. Conversely, staphylokinase promotes bacterial spread by dissolving fibrin clots through plasminogen activation (Tam and Torres, 2019).

Furthermore, capsular polysaccharides (primarily types 5 and 8) provide resistance against phagocytosis, while catalase and the carotenoid pigment staphyloxanthin protect MRSA against oxidative stress by neutralizing reactive oxygen species.

### 4.2 Extracellular toxins

#### 4.2.1 Staphylococcal enterotoxins

*S. aureus* produces a wide range of staphylococcal enterotoxins (SEs), which function as potent superantigens. This group includes at least 25 members, comprising classical SEs and enterotoxin-like (SEl) proteins (Lefebvre et al., 2022). These toxins bind to MHC class II molecules on antigen-presenting cells and T-cell receptors, leading to massive, non-specific T-cell activation and excessive cytokine release. This hyperactivation results in severe clinical conditions such as staphylococcal food poisoning and toxic shock syndrome (TSS; Tristan et al., 2007).

Toxic Shock Syndrome Toxin-1 (TSST-1), closely related to the SE family, is a major virulence factor associated with both menstrual and non-menstrual TSS, often leading to life-threatening systemic effects (Thacharodi et al., 2026).

#### 4.2.2 Hemolysins

MRSA produces several hemolysins, including *α*-, *β*-, *γ*-, and *δ*-toxins, which contribute to host cell damage. Among these, α-hemolysin is the most significant and is produced by the majority of pathogenic strains of MRSA and MSSA. It forms heptameric transmembrane pores in host cell membranes, leading to cell lysis and tissue necrosis. This toxin is particularly associated with severe infections, including pneumonia and tissue destruction (Shettigar and Murali, 2020).

Additionally, certain MRSA strains harbor bacteriophages such as *Φ*-PVL, which encode Panton-Valentine leukocidin (PVL; Lina et al., 1999). PVL is a bicomponent toxin (LukS-PV and LukF-PV) that forms pores in neutrophils and epithelial cells, leading to cell death. PVL-positive strains are strongly associated with severe clinical manifestations such as necrotizing skin infections, necrotizing pneumonia, and osteomyelitis.

#### 4.2.3 Exfoliative toxins

Exfoliative toxins (exfoliatins A and B) are serine protease enzymes responsible for Staphylococcal Scalded Skin Syndrome (SSSS). This condition primarily affects infants and young children. These toxins target desmoglein-1 within the epidermis, leading to a loss of cell adhesion and subsequent peeling of the skin. SSSS can also occur in hospital settings particularly in neonatal units and has the potential to spread in the form of outbreaks (Kong et al., 2016).

Overall, the virulence of MRSA is a multifaceted characteristic governed by the coordinated interplay of structural components, adhesion factors, immune evasion strategies, and potent toxins. This complex mechanism enables MRSA to successfully colonize, persist within a host, and cause severe infections.

## 5 Biofilm state of the *Staphylococcus aureus*

Biofilm formation in *S. aureus* is a highly regulated, multi-step process, primarily governed by the Agr quorum-sensing system. The initial formation of the biofilm is facilitated by the suppression of the Agr system, resulting in increased production of surface adhesins. These elements enable the biofilm to attach to both biotic and abiotic surfaces. This is followed by bacterial aggregation, proliferation, and the production of extracellular polymeric substances (EPS), leading to the maturation of a structured biofilm. In a subsequent phase, the reactivation of the Agr system triggers the synthesis of proteases, hydrolases, and autolysins, resulting in the partial lysis of cells and the release of extracellular DNA (eDNA). This eDNA enhances the structural stability of the biofilm and facilitates the dispersal of cells into a planktonic state, thereby further propagating the infection (Cheng et al., 2023; Wu et al., 2024).

*S. aureus* biofilms can be broadly categorized into PIA-dependent, PIA-independent, and eDNA-mediated types, based primarily on their matrix composition. PIA-dependent biofilms typically associated with HA-MRSA, rely on the synthesis of poly-N-acetylglucosamine (PIA/PNAG) mediated by the icaADBC operon (Chopra et al., 2015; Xu L. et al., 2025). This operon governs the processes of intercellular adhesion and surface attachment. The icaADBC operon is regulated by various factors such as SarA, SigB, SrrAB, and Rbf, as well as by repressor elements like IcaR and CodY (Mlynarczyk-Bonikowska and Rudnicka, 2025).

Conversely, PIA-independent biofilms develop in environments where the ica operon is inactive. The development of these biofilms necessitates cell aggregation and attachment to host matrix components, facilitated by surface-associated proteins such as FnBPs, ClfA, Spa, and SasG (Lei et al., 2011). Furthermore, eDNA-mediated biofilms arise from autolysis driven by the cidABC operon and regulated by the reduced expression of SarA and *nuc*. The released DNA contributes to the integrity of the matrix, the modulation of surface charge, and the stabilization of the biofilm (Hirschhausen et al., 2012; Porayath et al., 2018).

In both MSSA and MRSA, biofilm formation occurs through distinct genetic mechanisms. In MRSA, the process of biofilm formation is ica-independent and is mediated by surface-associated proteins. The *mecA* gene present in MRSA enhances the biofilm formation process by inactivating the accessory gene regulator quorum-sensing system, the very system that also governs the virulence of *S. aureus* (Regassa et al., 1992; Fournier and Hooper, 2000). The presence of SCCmec elements in MRSA suppresses colony spreading activity, downregulates the expression of chromosomally encoded PSMα, and thereby attenuates virulence (Novick and Subedi, 2007). Several studies have reported that MRSA exhibits a higher rate of biofilm formation, a phenomenon attributed to the underlying genetic mechanisms involved. Conversely, some studies have also observed a higher prevalence of biofilm formation in MSSA.

The biofilm mode of growth confers several unique features that distinguish it from the planktonic state and significantly impact disease progression. *S. aureus* can exist in either a planktonic (free-floating) or a biofilm-associated form (Mirani et al., 2017). Planktonic cells may be associated with acute infections and can readily multiply and disseminate throughout the host (body). Conversely, chronic infections may involve biofilms, wherein *S. aureus* cells are entrapped within an extracellular, protective polysaccharide-protein-extracellular DNA matrix. In this structure, proteins associated with the cell membrane form and interact with the matrix (Archer et al., 2011; Marimuthu et al., 2026). *S. aureus* is capable of thriving on various surfaces within healthcare environments, such as medical devices (surgical instruments, implants, etc.; Weber et al., 2023). Unless properly sanitized and controlled, biofilms associated with such surfaces and medical devices can pose a serious infection risk to patients. In particular, biofilms formed on implanted devices often necessitate their removal and replacement, thereby further increasing surgical risks and complications (Daubert and Weinstein, 2019). Conversely, acute infections caused by planktonic cells can progress rapidly, potentially resulting in severe illness.

Bacteria are capable of switching their mode of growth between planktonic (single-cell) and biofilm forms. They colonize solid surfaces and firmly embed themselves within bacterial communities residing within an extracellular polymeric matrix. It has been observed that biofilm bacteria are a thousand times more resistant to conventional medications than planktonic bacteria, and they also withstand attacks from the host’s immune system more robustly (Rehal et al., 2017).

## 6 Mechanisms of antibiotic resistance

**Figure 2:**
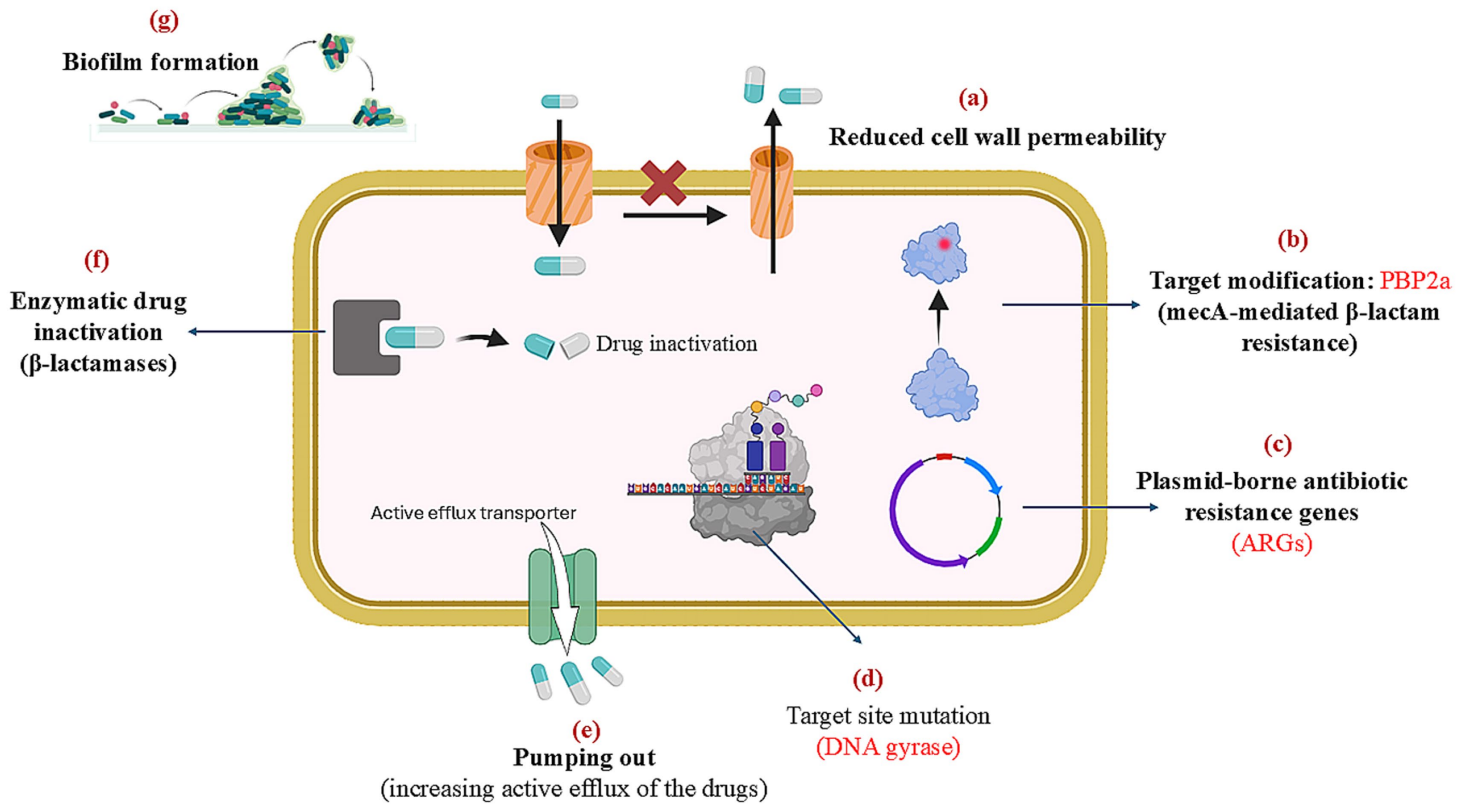
Schematic representation of the major antibiotic resistance mechanisms in Methicillin-resistant *Staphylococcus aureus*, including **(a)** reduced cell wall permeability, **(b)** target modification mediated by PBP2a (*mecA* gene), **(c)** plasmid-borne antibiotic resistance genes, **(d)** target-site mutations, **(e)** active efflux pumps, **(f)** enzymatic drug inactivation by β-lactamases, and **(g)** biofilm formation contributing to antimicrobial tolerance and persistence. Created with BioRender.com.

The evolution and emergence of MRSA are largely attributed to its capacity to acquire and develop resistance against a wide range of antimicrobial agents. This resistance is facilitated by several genetic and biochemical mechanisms that reduce antibiotic efficacy and promote bacterial survival. These mechanisms play a critical role in the persistence and dissemination of MRSA in both clinical and community settings. The major mechanisms of antibiotic resistance are summarized below and major molecular and biochemical mechanisms contributing to antibiotic resistance in MRSA are illustrated in Figure 2.

### 6.1 PBP2a-mediated β-lactam resistance in MRSA

Methicillin resistance in *S. aureus* arises primarily through acquisition of the *mecA* gene, which encodes the altered PBP2a with reduced affinity for β-lactam antibiotics (Otero et al., 2013; Anitha et al., 2016). Under normal conditions, β-lactams inhibit cell wall synthesis by mimicking the D-Ala–D-Ala substrate and covalently binding to native PBPs, thereby blocking the transpeptidation step required for peptidoglycan cross-linking (Kim et al., 2023). However, PBP2a retains transpeptidase activity even in the presence of *β*-lactams due to its low binding affinity and reduced acylation efficiency, allowing continued cell wall synthesis (Fisher and Mobashery, 2021). Structurally, this resistance is associated with a narrow and conformationally restricted active site, which limits antibiotic access. In addition, PBP2a is regulated by an allosteric mechanism, where ligand binding at a distal site induces conformational changes that transiently open the active site for catalysis (Rosado et al., 2025). Mutations affecting either the active or allosteric regions can further modulate resistance, highlighting the dynamic nature of *mecA*-mediated resistance (Pinho and Foster, 2024). Consistent to this mechanism, classical β-lactams, such as methicillin and oxacillin, are ineffective against MRSA because of impaired acylation of PBP2a. In contrast, newer cephalosporins, such as ceftaroline and ceftobiprole, overcome this limitation to some extent by targeting PBP2a; specifically, ceftaroline binds initially to the allosteric site and induces conformational opening of the active site for inhibition. However, resistance to these agents has already been reported and is primarily due to mutations in (e.g., N146K, N204K, G246E), which disrupt allosteric signalling and reduce drug binding (Schaumburg et al., 2016; Morroni et al., 2018). Overall, PBP2a-mediated resistance in MRSA is driven by reduced β-lactam binding, structural inaccessibility of the active site, and allosteric control, which together sustain peptidoglycan synthesis despite antibiotic exposure.

### 6.2 Mec-independent methicillin resistance in *Staphylococcus aureus*

Resistance to β-lactam antibiotics in *S. aureus* is typically mediated by PBP2a, which is encoded by the *mecA* gene or its homologs *mecB* and *mecC* (Becker et al., 2018). However, some methicillin-resistant strains lack these known *mec* genes and are therefore classified as *mec*-independent methicillin-resistant (MRLM) strains. To investigate the genetic basis of this resistance, comparative genomic and genome-wide association studies (GWAS) were performed on 141 MRLM isolates and 142 methicillin-susceptible controls. These analyses revealed a strong association between the MRLM phenotype and mutations in the *gdpP* gene, which encodes a phosphodiesterase responsible for degrading the bacterial second messenger cyclic-di-AMP (Sommer et al., 2021).

**Figure 3:**
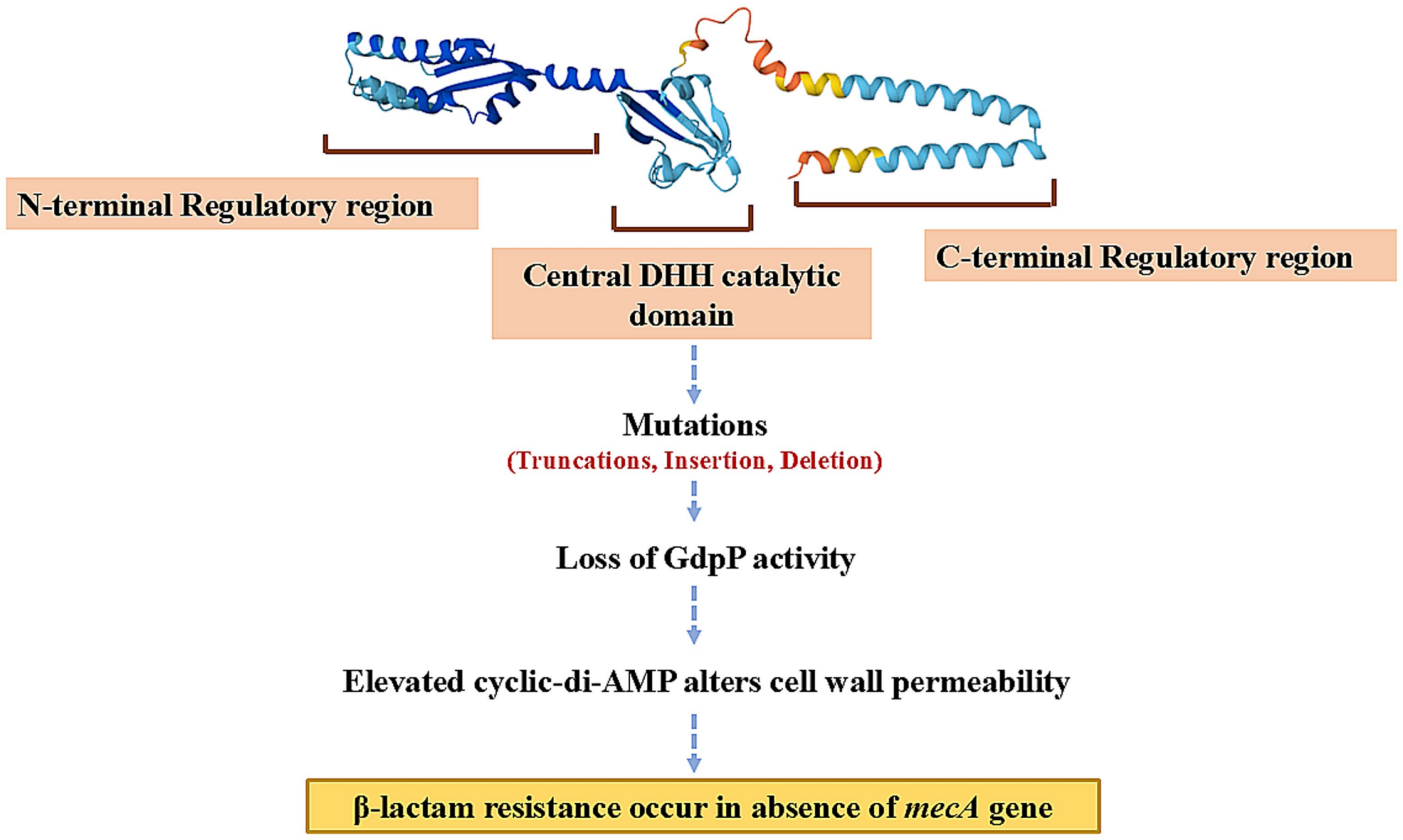
Schematic representation of GdpP protein domain organization and the role of *gdpP* mutations in mediating *mecA*-independent β-lactam resistance in *Staphylococcus aureus* infection. Mutations within the central DHH catalytic domain led to loss of GdpP activity, elevated cyclic-di-AMP levels, altered cell wall permeability, and subsequent β-lactam resistance in the absence of the *mecA* gene.

Most MRLM isolates contained truncations, insertions, deletions, or amino acid substitutions within the catalytic DHH domain of GdpP (Figure 3). Complementation studies confirmed that these mutations directly contribute to *β*-lactam resistance. Although *pbp4* transcription remained unaffected, the mutant strains exhibited reduced cell size and strain-specific variations in biofilm formation (Ba et al., 2019).

In addition to *gdpP*-mediated resistance, recent genomic studies have identified alternative non-*mecA* mechanisms associated with reduced susceptibility to advanced β-lactams. Whole-genome sequencing of ceftaroline- and ceftobiprole-resistant *S. aureus* strains revealed recurrent mutations upstream of the *pbp4* gene and within the PBP4 protein itself, particularly at residue Asn138. These alterations are thought to enhance PBP4 activity or expression, thereby contributing to β-lactam resistance independently of PBP2a. Furthermore, mutations in regulatory and stress-response genes, including *clpX*, PP2C phosphatase, and transcription terminator *rho*, were also identified, suggesting a broader adaptive network underlying resistance (Greninger et al., 2016).

Collectively, these findings highlight *gdpP* mutations as a clinically relevant, *mec*-independent mechanism of β-lactam resistance in *S. aureus*, underscoring the increasing complexity of resistance pathways beyond conventional *mec*-mediated systems (Sommer et al., 2021).

### 6.3 Target site mutations contributing to antibiotic resistance in MRSA

Mutation-driven alterations in ribosomal and transcription-associated machinery represent an important mechanism of antibiotic resistance in MRSA (Gomez et al., 2017). In recent study, *in vitro* selection of linezolid-resistant mutants from cfr-negative MRSA isolates demonstrated that resistance to oxazolidinones is primarily mediated by chromosomal mutations rather than acquired genes. This study identified mutations in the *rplC* gene encoding the 50S ribosomal protein L3 and the *rpoB* gene encoding the β-subunit of RNA polymerase, where the Gly152Asp substitution in L3 was shown to confer cross-resistance to both linezolid and tedizolid, likely through structural alterations at the peptidyl transferase center. Additionally, mutations in efflux-associated genes such as *mepB*, part of the *mepRAB* operon, have been suggested to enhance resistance through altered regulatory control and increased drug efflux. Collectively, these studies highlight that mutation-mediated resistance in MRSA involves multiple independent yet interconnected mechanisms, including ribosomal modification, transcriptional regulation, and efflux modulation, contributing to variable resistance phenotypes and cross-resistance across antibiotic classes (Goda et al., 2025). In another study, serial passage experiments identified a novel *rpoB* mutation (D449N) associated with reduced susceptibility to tedizolid and other PhLOPSa antibiotics, suggesting that transcriptional alterations can indirectly mediate cross-resistance without directly affecting the antibiotic binding site (Shen et al., 2021). In one more clinical study, resistance to linezolid was primarily associated with mutations in the 23S rRNA gene, particularly the T2500A substitution, whereas the L3 Gly152Asp mutation was frequently detected in susceptible isolates, indicating that its contribution to resistance may be context-dependent (Yoo et al., 2020). These findings underscore that mutation-driven resistance in MRSA is multifactorial and highly adaptive, arising from coordinated changes in ribosomal targets, transcriptional machinery, and regulatory pathways that collectively reduce antibiotic efficacy.

### 6.4 Efflux pump-mediated resistance

Efflux pump-mediated resistance represents a critical non-target-based mechanism contributing to multidrug resistance in *S. aureus*, particularly in MRSA strains. These membrane-embedded, energy-dependent transporters actively expel a wide range of structurally diverse antibiotics, including fluoroquinolones, tetracyclines, and macrolides, thereby reducing intracellular drug concentrations below therapeutic levels. Major efflux systems such as NorA, NorB, and NorC (MFS family) play a dominant role in fluoroquinolone resistance, while Tet(K)/Tet(L) and QacA/B pumps contribute to resistance against tetracyclines and antiseptics, respectively. Both chromosomally encoded and plasmid-mediated pumps collectively enhance bacterial survival under antibiotic stress. Importantly, the overexpression of these pumps, regulated by global transcriptional regulators such as MgrA and NorG, further amplifies resistance and facilitates adaptive responses to antimicrobial exposure. In the context of our study, such efflux-driven mechanisms likely act synergistically with target-site mutations (e.g., in gyrA/gyrB or PBP variants), thereby strengthening resistance phenotypes and complicating therapeutic outcomes (Costa et al., 2013). Therefore, efflux pump activity not only reduces drug efficacy but also represents a promising target for adjunct therapeutic strategies such as efflux pump inhibitors to restore antibiotic susceptibility (Ojha and Beuria, 2026).

### 6.5 Biofilm-mediated antibiotic resistance

Biofilm formation is a major mechanism by which MRSA enhances its survival, persistence, and resistance to antimicrobial therapy. Biofilms are structured microbial communities embedded in an extracellular polymeric substance (EPS) matrix composed of polysaccharides, proteins, and extracellular DNA, which acts as a protective barrier against antibiotics and host immune responses (Idrees et al., 2021). Environmental factors such as sub-inhibitory antibiotic levels and stress conditions further promote biofilm development (Kaushik et al., 2024).

Biofilm formation occurs in stages, including initial attachment, maturation, and dispersion (Silva et al., 2021). The initial adhesion to surfaces is mediated by MSCRAMMs such as ClfA and ClfB, along with other adhesins that facilitate colonization (Ghasemian et al., 2015). During maturation, the EPS matrix is formed, where polysaccharide intercellular adhesin (PIA), encoded by the *icaADBC* operon, plays a key role, although *ica*-independent mechanisms also contribute (Maira-Litrán et al., 2002).

The EPS matrix significantly contributes to resistance by limiting antibiotic penetration and promoting reduced metabolic activity, leading to decreased susceptibility (Di Martino, 2018). Additionally, biofilms protect bacteria from immune clearance. In the final stage, dispersion of biofilm cells, regulated by the *agr* quorum-sensing system, enables bacterial spread and recurrent infections (Wille and Coenye, 2020).

### 6.6 Other mechanisms of antibiotic resistance

In addition to mutation-driven, efflux-mediated, genetic transfer, and biofilm-associated resistance, *S. aureus* employs several additional mechanisms to withstand antimicrobial pressure. One important strategy involves the formation of persister cells, a subpopulation capable of surviving lethal stress and resuming growth after antibiotic exposure, thereby contributing to persistent and recurrent infections (Lewis, 2010). Moreover, adaptive phenotypic variations such as small colony variants further enhance bacterial survival under hostile conditions (Hernando-Amado et al., 2019; Bowler et al., 2020).

Intrinsic resistance also plays a significant role, arising from the inherent biological characteristics of the organism rather than acquired genetic elements. These mechanisms include reduced membrane permeability, lack of affinity between antibiotic and its target, inability of the drug to enter the bacterial cell, active efflux of antibiotics, and enzymatic degradation of antimicrobial agents (Martínez, 2018).

Additionally, horizontal gene transfer facilitates the spread of antimicrobial resistance genes across bacterial populations, enabling the transition of resistance traits from environmental and commensal organisms to pathogenic strains, thereby expanding the resistome (Deurenberg and Stobberingh, 2008; Dey et al., 2020; Di Donato et al., 2025). Among the mechanisms of gene transfer, conjugation is considered the most effective due to its efficiency and broader host range (Norman et al., 2009).

Collectively, these mechanisms function by either reducing the effective concentration of antibiotics reaching their target or by altering the target itself, including key enzymes such as DNA gyrase involved in replication processes. Processes such as decreased permeability, enhanced efflux, enzymatic inactivation, and target modification act in combination to compromise antibiotic efficacy and promote bacterial survival under antimicrobial stress (Christaki et al., 2020; Zhu et al., 2022).

## 7 Therapeutic sites in MRSA

The diverse mechanisms of antibiotic resistance in MRSA significantly limit the efficacy of conventional antimicrobial therapies. These resistance strategies not only enhance bacterial survival but also complicate treatment outcomes in clinical settings. Consequently, there is a growing need to identify and explore alternative molecular targets that can overcome or bypass these resistance mechanisms. In this context, several therapeutic targets have been investigated in MRSA, which are discussed below.

### 7.1 Iron acquisition pathways as therapeutic targets

Iron is an essential nutrient for the growth, metabolism, and virulence of *S. aureus*. During infection, the host restricts iron availability through a process known as nutritional immunity, in which iron is tightly sequestered by proteins such as transferrin, lactoferrin, and hemoglobin (Weinberg, 1975). To overcome this limitation, *S. aureus* has evolved efficient iron acquisition systems, making these pathways attractive therapeutic targets (Conroy et al., 2019).

*S. aureus* acquires iron primarily through two complementary mechanisms: siderophore-mediated iron uptake and heme acquisition. The bacterium synthesizes two siderophores, staphyloferrin A and staphyloferrin B, which chelate ferric iron (Fe^3+^) from the surrounding environment. These iron-loaded complexes are transported into the bacterial cell by specific ATP-binding cassette (ABC) transporters, while additional transport systems enable the uptake of siderophores produced by other microorganisms (Konetschny-Rapp et al., 1990; Haag et al., 1994; Davidson et al., 2008). Alternatively, *S. aureus* utilizes the iron-regulated surface determinant (Isd) system to extract heme from host hemoglobin, transport it into the cytoplasm, and release iron through heme degradation. Together, these pathways enable the bacterium to survive and proliferate under iron-limited conditions encountered during infection (Mazmanian et al., 2003; Abe et al., 2012).

Given their essential role in bacterial survival and pathogenicity, iron acquisition systems have become promising targets for the development of new anti-MRSA therapies. Current strategies include inhibiting siderophore biosynthesis, targeting Isd proteins and siderophore receptors through vaccine development, and exploiting bacterial iron uptake pathways for drug delivery. In the “Trojan horse” strategy, antibiotics are covalently linked to siderophores, allowing the conjugates to utilize bacterial iron transport systems for active intracellular uptake. Once inside the bacterial cell, the antibiotic is released to exert its antibacterial effect, thereby enhancing drug delivery and potentially improving therapeutic efficacy (Milner et al., 2013). Although this approach has shown greater success against Gram-negative bacteria, it is also being investigated as a promising strategy for *S. aureus* infections.

Overall, targeting iron acquisition pathways offers an attractive antivirulence strategy by depriving *S. aureus* of an essential nutrient while simultaneously improving the effectiveness of antimicrobial therapy. Continued research into these pathways may facilitate the development of targeted therapeutics against multidrug-resistant MRSA.

### 7.2 SaeR as a target for anti-virulence drug development

Deletion of *SaeR* in *S. aureus* has been shown to reduce the occurrence of skin infection, pneumonia, bacteraemia, and virus–MRSA co-infection (Borgogna et al., 2018). This reduction in virulence facilitates clearance of infection by the host immune system. Anti-virulence strategies are advantageous because they suppress pathogenicity without imposing strong selective pressure and help preserve the host microbiota (Gao et al., 2023).

*SaeR* is a response regulator of the *saeRS* two-component system and functions as a downstream regulator controlling the expression of multiple virulence-associated genes (Cheng et al., 2009). It modulates the expression of surface proteins such as Spa, FnbA, and Emp, as well as secreted proteins including SspA, HlgA, CHIPS, and SplA. In addition, *SaeR* regulates metabolic pathways such as purine synthesis, pyrimidine biosynthesis, and the arginine deiminase pathway (Voyich et al., 2009).

Inhibition of *SaeR* leads to reduced expression of virulence factors such as HlgA and HlgC, which are involved in *γ*-haemolysin production, thereby decreasing bacterial survival in the bloodstream (Malachowa and DeLeo, 2011). The chemotaxis inhibitory protein of *S. aureus* (CHIPS), encoded by *chp*, is an exoprotein that inhibits neutrophil and monocyte chemotaxis and enhances bacterial survival. Suppression of CHIPS expression exposes bacteria to host immune responses and reduces bacterial load during infection (van Wamel et al., 2006).

The study identified HR3744 as a small-molecule inhibitor of *SaeR* using a dual GFP-lux reporter system driven by the *saeP1* promoter. HR3744 inhibited the interaction between phosphorylated *SaeR* and DNA, as demonstrated by electrophoretic mobility shift assay, and directly interacted with the DNA-binding domain of *SaeR*, as confirmed by WaterLOGSY-NMR analysis. The inhibitor reduced the expression of virulence genes such as *hla*, *pvl*, *psm*, and *agr*, and decreased haemolytic and leucotoxic activity without affecting bacterial growth. Target specificity was confirmed through genetic and mutagenesis studies. HR3744 lost its activity in *saeR* deletion strains, indicating that its anti-virulence effect is dependent on *SaeR*. Site-directed mutagenesis showed that substitution of glutamic acid at position 159 (E159N) reduced sensitivity to HR3744, demonstrating that this residue is important for inhibitor binding. A structurally similar analogue, HR3763, lacked inhibitory activity, further confirming specificity. Structure–activity relationship analysis identified SAV13 as a more potent analogue of HR3744, with approximately four- to fivefold higher anti-virulence activity. SAV13 demonstrated similar target specificity and effectively reduced virulence gene expression in both *in vitro* assays and a mouse bacteraemia model (Gao et al., 2023).

### 7.3 Ribosomal A-site as a therapeutic target

Targeting nucleic acids with small molecules has been recognized as an effective therapeutic approach against bacterial pathogens, since these compounds can disrupt RNA-mediated processes and interfere with translation. The bacterial ribosome is a major target for such therapeutics, as it plays a central role in protein synthesis by translating genetic information into functional proteins (Steitz, 2008).

The ribosomal A-site, located within the decoding center of the 30S subunit and primarily formed by the 16S rRNA, is responsible for monitoring the correct base pairing between mRNA codon and aminoacyl-tRNA (aa-tRNA) anticodon (Dao et al., 2018). This region includes highly conserved nucleotides such as A1492, A1493, and G530, which undergo conformational rearrangements to ensure translational fidelity during protein synthesis (Ogle et al., 2001).

Several classes of antibiotics specifically target the ribosomal A-site and disrupt normal translation. Tetracyclines bind to the decoding center and inhibit protein synthesis by preventing the accommodation of aa-tRNA into the A-site, thereby blocking elongation (Jenner et al., 2013). In contrast, negamycin binds to an overlapping site but stabilizes tRNA binding in the A-site, leading to inhibition of translocation and induction of translational errors.

Aminoglycosides, such as paromomycin and gentamicin, represent another important class of A-site targeting antibiotics. These compounds bind to helix 44 (h44) of the 16S rRNA and induce conformational changes in nucleotides A1492 and A1493, promoting a flipped-out conformation that leads to misreading of the genetic code and incorporation of incorrect amino acids (Carter et al., 2000). Similarly, streptomycin interacts with regions surrounding the decoding center, including h18, h27, and ribosomal protein uS12, thereby altering the accuracy of translation (Demirci et al., 2013).

Other antibiotics, such as hygromycin B and viomycin, also interact with the A-site region and influence ribosomal dynamics. Hygromycin B increases the affinity of tRNA for the A-site and inhibits translocation, whereas viomycin stabilizes the ribosome in a specific conformational state that prevents proper progression of translation (Borovinskaya et al., 2008).

Structurally, the A-site region is characterized by a conserved stem–loop architecture within helix 44 of the 16S rRNA, which provides a defined binding pocket for small molecules. These interactions are typically mediated through hydrogen bonding, electrostatic interactions, and stacking interactions between antibiotic molecules and rRNA nucleotides, enabling high-affinity binding and effective inhibition of protein synthesis (Lin et al., 2018).

### 7.4 Micrococcal nuclease (MNase) as a virulence-associated therapeutic target

Targeting bacterial virulence factors rather than essential survival pathways has emerged as a promising strategy to combat antimicrobial resistance. Micrococcal nuclease (MNase), a secreted enzyme produced by *S. aureus*, plays a crucial role in immune evasion by degrading neutrophil extracellular traps (NETs), thereby facilitating bacterial survival and dissemination (Wiemels et al., 2021). Consequently, MNase represents an attractive therapeutic target for the development of anti-virulence agents. In one study, a naphthalimide-based ligand (C1) was identified as an effective inhibitor of MNase, a key virulence factor in MRSA. The synthetic naphthalimide-based ligands (C1–C3) were selected based on their physicochemical properties, including lipophilicity and the presence of hydrogen bond acceptor groups, which are recognized as important features in nuclease inhibitors (Sahareen et al., 2018). In addition to nuclease activity, the ability of *S. aureus* to adhere to host tissues is a critical factor in its pathogenesis, particularly in collagen-rich tissues such as bone, cartilage, and skin (Elasri et al., 2002). This adhesion is mediated by staphylococcal collagen adhesin (Cna), a major virulence factor involved in MRSA colonization, biofilm formation, and tissue invasion. Therefore, inhibition of collagen adhesion represents a potential strategy to prevent MRSA-associated infections, especially those related to implanted medical devices (Pecoraro et al., 2023).

Previous studies have shown that triblock copolymers, such as PF-127, and certain amphiphilic ligands can reduce staphylococcal adhesion to biomaterial surfaces (Dey et al., 2020). Building on this concept, a PF-127-based micellar nanocarrier (C1-PMC) loaded with the hydrophobic ligand C1 was developed to enhance anti-adhesion activity. Experimental results demonstrated that C1 reduced MRSA adhesion in a dose-dependent manner in collagen adhesion assays, whereas C1-PMC exhibited significantly enhanced inhibitory effects (Konwar et al., 2024). Mechanistic studies revealed that C1 acts as a non-competitive inhibitor of MNase, reducing the enzyme’s turnover number and catalytic efficiency. Furthermore, *in vitro* analysis showed that C1 enhances uptake in activated macrophage-like cells, promoting the entrapment of MRSA within DNA structures and thereby inhibiting MNase-mediated degradation (Kolarević et al., 2019).

Overall, these studies demonstrate that MNase inhibition, particularly through naphthalimide-based ligands such as C1 and its nanocarrier formulation, can effectively reduce enzymatic activity and bacterial adhesion. These findings highlight the functional importance of MNase in *S. aureus* pathogenicity and illustrate how its inhibition can modulate virulence-associated processes.

## 8 Clinically approved chemical therapeutics against MRSA

Antibiotic therapy plays a central role in the current treatment of MRSA infections. Building upon the identified therapeutic targets in MRSA, several clinically approved synthetic antibiotics have been developed that specifically interfere with key bacterial processes such as cell wall biosynthesis and protein synthesis. These agents act on well-defined molecular targets and remain central to the treatment of MRSA infections. Depending on the severity and symptoms of the infection, these antibiotics may be administered as monotherapy or combination therapy.

Among synthetic antibiotics, methicillin and penicillin were initially used for the treatment of *S. aureus* infections, although resistance rapidly limited their effectiveness. Vancomycin remains a cornerstone drug for MRSA treatment and acts by inhibiting peptidoglycan biosynthesis through strong binding to the D-Ala-D-Ala termini of cell wall precursors (Belley et al., 2010). Daptomycin, a cyclic lipopeptide, exerts its bactericidal activity by disrupting the bacterial cell membrane in a concentration-dependent manner and has shown activity comparable to or greater than vancomycin and linezolid *in vitro* (Carpenter and Chambers, 2004). However, vancomycin and daptomycin remain the first-line agents for the treatment of MRSA bacteremia, while rifampicin is frequently added in cases of implant-associated infections because of its excellent biofilm penetration (Zimmerli and Sendi, 2019).

**Figure 4:**
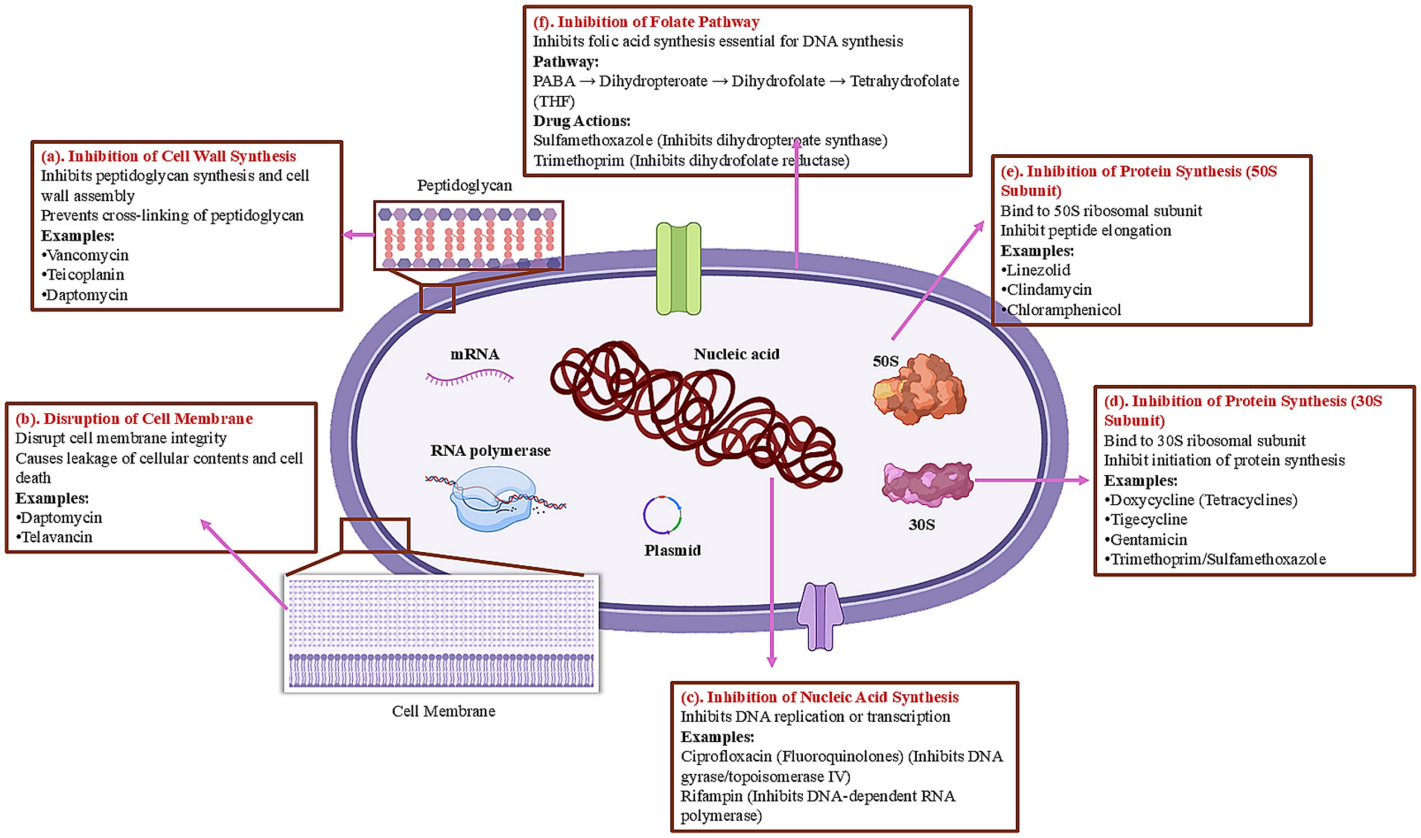
Schematic representation of the major therapeutic targets and mechanisms of action of existing antibiotics against methicillin-resistant *Staphylococcus aureus* (MRSA). The figure illustrates antibiotics targeting **(a)** inhibition of cell wall synthesis, **(b)** disruption of the cell membrane, **(c)** inhibition of nucleic acid synthesis, **(d)** inhibition of protein synthesis (30S ribosomal subunit), **(e)** inhibition of protein synthesis (50S ribosomal subunit), and **(f)** inhibition of the folate biosynthesis pathway. Created with BioRender.com.

Mupirocin is another clinically important agent that inhibits bacterial protein synthesis by targeting isoleucyl tRNA synthetase (Hughes and Mellows, 1978). The oxazolidinone linezolid has broad-spectrum activity against MRSA, methicillin-resistant *S. epidermidis* (MRSE), and VRSA, acting by inhibiting the initiation of protein synthesis (Song, 2008). Alternative treatment options include drugs such as, ceftaroline, ceftobiprole, lipoglycopeptides (dalbavancin, oritavancin, and telavancin), linezolid, and tedizolid. These agents are effective in inhibiting essential bacterial processes, such as cell wall synthesis or protein synthesis (Table 1). The major therapeutic targets and mechanisms of action of currently available antibiotics against MRSA are illustrated in Figure 4.

**Table 1:** Major antibiotics used against MRSA and their corresponding molecular targets and mechanisms of action. The table summarizes antibiotics targeting cell wall synthesis, protein synthesis, membrane integrity, and DNA replication.

Class	Antibiotic	Target (function)	Mode of action
β-Lactams	Ampicillin, amoxicillin, methicillin	Penicillin-binding proteins (PBPs) involved in peptidoglycan synthesis	Inhibits transpeptidation leading to defective cell wall and lysis (Rolinson, 1998)
β-Lactams + β-lactamase inhibitors	Amoxicillin + clavulanate	Penicillin-binding proteins (PBPs); β-lactamase enzymes	Amoxicillin inhibits PBPs, blocking peptidoglycan cross-linking and cell wall synthesis, while clavulanate irreversibly inhibits β-lactamases, protecting amoxicillin from enzymatic degradation (Rolinson, 1998)
Glycopeptides	Vancomycin, teicoplanin	D-Ala-D-Ala termini of peptidoglycan precursors (lipid II)	Inhibits cell wall biosynthesis by preventing cross-linking (Reynolds, 1989)
Lipopeptide	Daptomycin	Bacterial cell membrane	Disrupts membrane integrity causing depolarization and cell death (Schriever et al., 2005)
Pseudomonic acid	Mupirocin	Isoleucyl-tRNA synthetase	Inhibits protein synthesis by blocking tRNA charging (Thomas et al., 2010)
Oxazolidinones	Linezolid, tedizolid (oxazolidinone)	50S ribosomal subunit (23S rRNA)	Inhibits initiation of protein synthesis (Moellering, 2003; Zhanel et al., 2015)
Macrolides	Erythromycin, azithromycin, roxithromycin, clarithromycin	50S ribosomal subunit (peptidyl transferase center/exit tunnel)	Inhibits protein elongation (Champney and Burdine, 1995)
Tetracyclines	Tetracycline, doxycycline, minocycline	30S ribosomal subunit (A-site)	Blocks aminoacyl-tRNA entry → inhibits protein synthesis (Pearson et al., 2025)
Aminoglycosides	Streptomycin, gentamicin, amikacin, arbekacin	30S ribosomal subunit (decoding center)	Causes misreading of mRNA and defective proteins (Magnet and Blanchard, 2005; Matsumoto, 2014)
Amphenicols	Chloramphenicol	50S ribosomal subunit (peptidyl transferase center)	Inhibits peptide bond formation (Fayyaz et al., 2013)
Fluoroquinolones	Ciprofloxacin, levofloxacin, moxifloxacin, delafloxacin	DNA gyrase and topoisomerase IV	Inhibits DNA replication (Gade and Qazi, 2013)
Glycylcyclines	Tigecycline	30S ribosomal subunit	Blocks tRNA binding and protein synthesis (Kasbekar, 2006)
Streptogramins	Oritavancin/dalbavancin	Cell wall synthesis (lipid II and peptidoglycan)	Inhibits cell wall biosynthesis and membrane integrity (Mast and Wohlleben, 2014)
Rifamycins	Rifampicin	DNA-dependent RNA polymerase	Inhibits RNA synthesis (Floss and Yu, 2005)
Antifolates	Trimethoprim–sulfamethoxazole	Dihydrofolate reductase (DHFR) and dihydropteroate synthase (DHPS)	Sequential inhibition of folate biosynthesis, resulting in impaired DNA synthesis (Cadena et al., 2011)

Although vancomycin remains the cornerstone of MRSA therapy, its clinical use is associated with several limitations, including the need for prolonged intravenous administration, therapeutic drug monitoring, nephrotoxicity, and poor oral bioavailability (Davis et al., 2016). Several newer antibiotics have been introduced to overcome these limitations and improve treatment outcomes. Nevertheless, most of these agents also require intravenous administration and may be associated with side effects. In addition, conventional antibiotics lack specificity and can cause dysbiosis by disrupting the normal microbiota. The widespread use of broad-spectrum antibiotics has led to the emergence of multidrug-resistant strains, including isolates exhibiting reduced susceptibility or cross-resistance to second-line therapies, thereby limiting available treatment options.

Combination antibiotic therapy has proven to be an effective strategy for improving treatment outcomes in persistent MRSA infections. Experimental and clinical studies have demonstrated synergistic interactions between vancomycin and other β-lactam antibiotics, showing that combination therapy clears MRSA bacteria more rapidly than vancomycin alone (Dilworth et al., 2014).

Similarly, combinations of daptomycin with β-lactams, particularly ceftaroline, have shown enhanced bactericidal activity against persistent and biofilm-producing MRSA isolates, while reducing the likelihood of resistance development (Kullar et al., 2011). Rifampicin is also widely used in combination with glycopeptides or β-lactams for prosthetic device-related infections because of its ability to penetrate biofilms and eradicate persister cells (Svensson et al., 1997).

The enhanced activity of glycopeptide or lipopeptide-based combinations has been partly attributed to the “see-saw effect.” This is an inverse relationship wherein reduced susceptibility to glycopeptides or lipopeptides leads to increased susceptibility to β-lactam antibiotics (Choo and Chambers, 2016). This is an inverse relationship wherein reduced susceptibility to glycopeptides or lipopeptides leads to increased susceptibility to β-lactam antibiotics (Vignaroli et al., 2011). Although the underlying mechanism is not yet fully understood, this phenomenon provides a rationale for combination therapy in treating difficult-to-manage MRSA infections. Despite these advances, the clinical pipeline for new anti-MRSA antibiotics remains limited, while drug resistance continues to rise (Tacconelli et al., 2018; Hajhamed et al., 2025). Therefore, the continuous development of new chemical entities, drug repurposing strategies, and combination therapies is essential to improve the long-term treatment and management of MRSA infections.

## 9 Novel therapeutic approaches against MRSA

**Figure 5:**
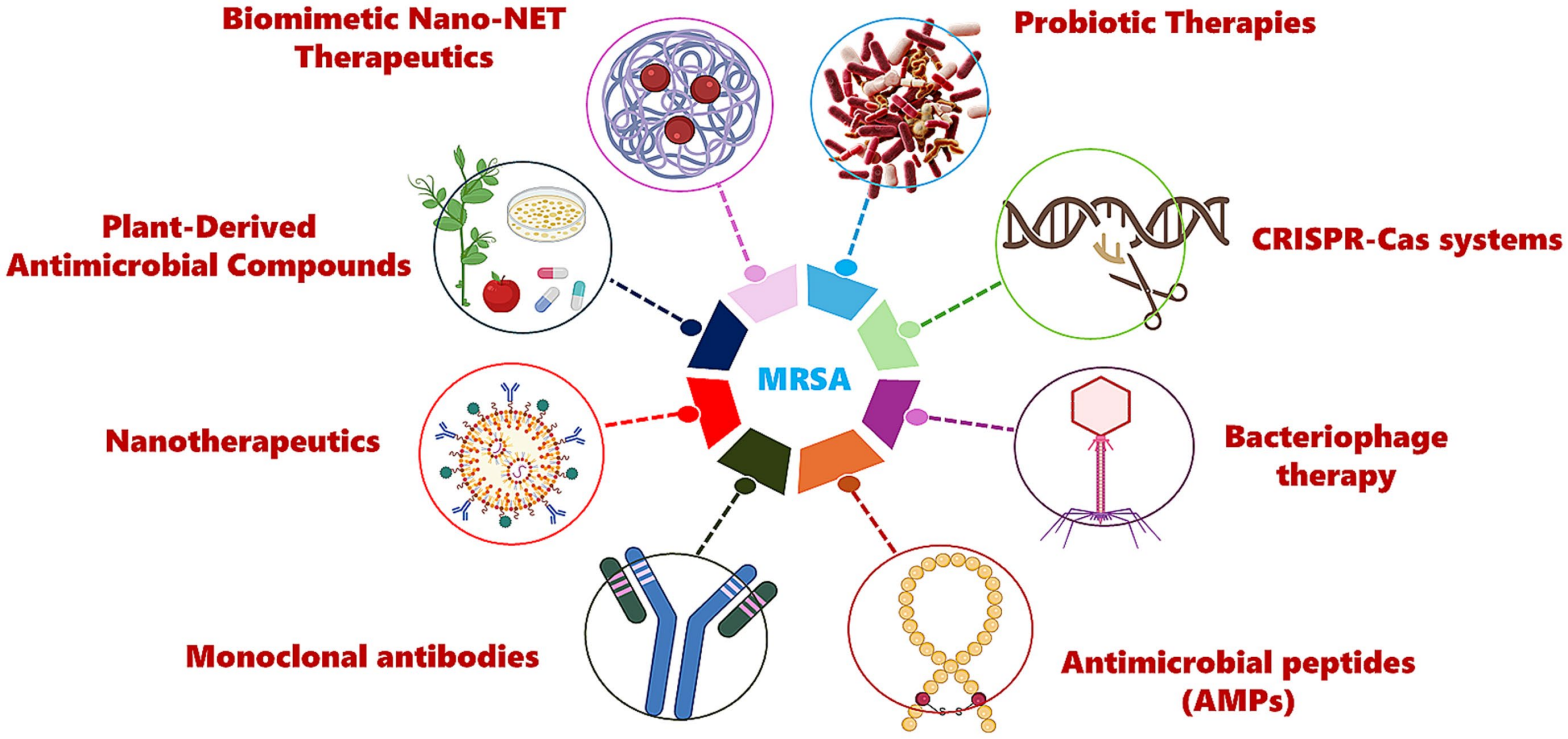
Emerging therapeutic strategies for combating methicillin-resistant *Staphylococcus aureus* (MRSA). Created with BioRender.com.

Despite the availability of several clinically approved antibiotics, the continuous emergence of MDR MRSA strains has significantly reduced the effectiveness of conventional therapies. Furthermore, the ability of MRSA to form biofilms, evade host immune responses, and acquire resistance determinants has complicated treatment outcomes and increased the risk of persistent and recurrent infections. These limitations have driven the exploration of innovative therapeutic strategies that can overcome resistance mechanisms and improve antibacterial efficacy. Therefore, the following sections discuss emerging therapeutic approaches and their potential applications for the effective management of MRSA infections. A schematic overview of these emerging therapeutic strategies against MRSA is presented in Figure 5.

### 9.1 Antimicrobial peptides as emerging therapeutics against MRSA

Antimicrobial peptides (AMPs) are small, cationic, and amphipathic molecules that generally consist of fewer than 50 amino acids. More than 5,000 AMPs have been identified to date. These peptides exhibit strong antibacterial activity primarily by disrupting bacterial membranes and also possess antibiofilm and immunomodulatory properties. Natural AMPs such as LL-37 play a crucial role in host defense, while larger proteins such as SPLUNC1 also demonstrate anti-biofilm activity against *S. aureus* (Britto and Cohn, 2015). Synthetic derivatives such as OP-145 show enhanced antimicrobial activity and have been reported to protect against biomaterial-associated *S. aureus* infections *in vivo* (de Breij et al., 2016).

Early studies demonstrated that MRSA can develop resistance to AMP through alterations in membrane charge, which reduces the binding of cationic peptides to the bacterial surface. However, this resistance can be overcome by combining AMPs with membrane-permeabilizing agents, which significantly enhances their bactericidal activity (Ravensdale et al., 2016).

Recent studies have focused on the rational and computational design of AMPs (Madni et al., 2024) reported an *in silico*-designed AMP that exhibited strong antibacterial and antibiofilm activity against MRSA without showing cytotoxic or hemolytic effects. Similarly, (Yuan et al., 2022) developed a novel peptide GW18 that demonstrated potent antibacterial activity against MRSA with minimal toxicity and significantly reduced bacterial infection in a mouse model.

Further advancements have led to the development of novel peptides with improved efficacy. Lin et al. (2025) reported a peptide named HfAMP that exhibited both bacteriostatic and bactericidal activity against MRSA. The study showed that HfAMP disrupts bacterial membrane integrity and significantly reduces bacterial burden and inflammation in a mouse skin infection model. In addition, Rühl-Teichner et al. (2025) demonstrated that AMPs such as Bac7(17) and PAsmr5-17 effectively inhibited bacterial growth and biofilm formation in MDR pathogens in a dose-dependent manner, achieving more than 99% growth inhibition at relatively low concentrations, thereby highlighting the broad-spectrum potential of AMPs against resistant bacteria.

Targeted antimicrobial strategies have also been developed to improve specificity. Xu B. et al. (2025) designed specifically targeted antimicrobial peptides (STAMPs), where the lead peptide P18E6 effectively disrupted MRSA cell membranes, eradicated biofilms, and demonstrated strong *in vivo* efficacy. The study also showed that combining STAMPs with bacterial-entrapping peptides further enhanced antibacterial activity and reduced systemic MRSA infections. Selected antimicrobial peptides with anti-*S. aureus* and anti-MRSA activity are given in Table 2.

**Table 2:** Antimicrobial peptides and peptide-based therapeutic agents investigated against MRSA and their corresponding antibacterial activities and therapeutic outcomes.

Agent	Type	Outcome
SPLUNC1 (Short Palate Lung Nasal Epithelial Clone 1)	Human airway protein (256 aa)	Exhibits anti-biofilm activity and contributes to host defense in the respiratory tract (Britto and Cohn, 2015)
LL-37	Antimicrobial peptide	Broad-spectrum activity; disrupts bacterial membrane; modulates host immune response against *S. aureus* (Haisma et al., 2014)
OP-145 (P60.4Ac) *(LL-37 derivative)*	Antimicrobial peptide	Protects against biomaterial-associated *S. aureus* infections; effective *in vivo* (rabbit model; de Breij et al., 2016).
Nisin	Antimicrobial peptide	Induces membrane permeabilization leading to bacterial cell death (Ganguly et al., 2025)
Human α-defensin	Antimicrobial peptide	Forms pores in bacterial membranes via channel-forming activity in lipid bilayers (Tai et al., 2015)
Human β-defensins (hBD-2, hBD-3)	Antimicrobial peptide	Strong antibacterial and immunomodulatory activity against *S. aureus* (Zhu et al., 2017)
Temporin-GHb3K	Antimicrobial peptide	Enhances wound healing; reduces bacterial burden and inflammation; decreases MRSA load in pneumonia model (Jin et al., 2026)
Temporin-GHbR	Antimicrobial peptide	Promotes MRSA wound healing (reduced CFU and cytokines); improves survival and reduces lung bacterial load (Jin et al., 2026)
Mastoparan X (MPX)	Antimicrobial peptide	Disrupts MRSA membrane, induces apoptosis-like death; prevents and eradicates biofilms (Lu et al., 2025)
HfAMP	Antimicrobial peptide	Stable across pH, temperature, and serum; significantly reduces CFU and inflammation in mouse MRSA skin infection model (Lin et al., 2025)
SAAP-148	Synthetic AMP	Rapid eradication of MRSA and biofilms, including persister cells (Scheper et al., 2021)
LTX-109	Synthetic tripeptide	Membrane disruption (Nilsson et al., 2015)
PLG0206	Engineered cationic antimicrobial peptide	Membrane disruption (Huang et al., 2022)
GSK1322322	Synthetic hydrazide	Peptide deformylase inhibitor (Butler et al., 2014)
Friulimicin B	Cyclic lipopeptide	Community acquired pneumonia staphylococcal skin infections (Schneider et al., 2009)
GW18	Synthetic AMP	Disrupts bacterial membrane integrity (Yuan et al., 2022)
Omiganan	Derivative of indolicidin	Disrupts bacterial membrane integrity, resulting in rapid bacterial killing (Wang et al., 2024)

Despite the promising therapeutic potential of AMPs, the possibility of resistance development should not be overlooked. Although AMPs generally exert rapid bactericidal activity and are considered less prone to resistance than conventional antibiotics, continuous or sub-inhibitory exposure may promote adaptive responses in susceptible bacteria. Bacteria can escape bacteriocins through both acquired and innate resistance mechanisms involving alterations in cell wall synthesis, cytoplasmic membrane composition, cell envelope architecture, membrane permeability, specific receptor expression, and energy metabolism or transport (Bastos et al., 2015; Andersson et al., 2016). In *S. aureus*, disruption of Dlt or MprF increases susceptibility to defensins, highlighting their role in modulating resistance to certain host-derived AMPs. Likewise, the BraRS two-component regulatory system controls the expression of the VraDE transporter, which contributes to resistance against nisin A, nukacin ISK-1, and bacitracin (Kawada-Matsuo et al., 2013; Randall et al., 2018), whereas the PmtA-D transporter, regulated by PmtR, is involved in resistance to *β*-defensin 3 and nisin A (Cheung et al., 2018). Furthermore, most evidence has been generated from laboratory selection studies under prolonged or sub-inhibitory peptide exposure, while clinically acquired resistance to therapeutic AMPs remains relatively uncommon. These observations highlight the need for rational peptide design and the use of combination therapies involving AMPs with different mechanisms of action or conventional antibiotics to reduce selective pressure and minimize the emergence of resistance (Bastos et al., 2015).

Overall, AMPs represent a promising class of next-generation therapeutics against MRSA due to their unique mechanisms of action, reduced likelihood of resistance development, and potential for rational design and targeted applications.

### 9.2 Nanomedicine-based therapeutic strategies against MRSA

Recently, nanomedicine has emerged as a promising avenue for combating MRSA infections particularly those associated with biofilms formation, which exhibit remarkable resistance to conventional antibiotics (Falagas et al., 2013). MRSA biofilms form a protective extracellular matrix, which can reduce antibiotic penetration and foster resistant populations, making their complete eradication difficult (Tarín-Pelló et al., 2022). Nanoparticle based approaches provides an effective option to overcome these challenges. They can penetrate and disturb the biofilms either by delivering the antibiotics directly into the matrix or by generating the reactive oxygen species. Metal based nanoparticles such as Ag, ZnO and CuO display antimicrobial activity by inducing oxidative stress, damaging cellular components and disrupt the membrane (Li et al., 2020; Makvandi et al., 2020). Furthermore, antimicrobial nanocoating based on metal nanoparticles, chitosan, or multifunctional polymer-drug composites have demonstrated anti-biofilm activity against MRSA. However, these multifunctional systems exhibit significant diversity, leading to major challenges regarding manufacturing and regulation, and complicating their use in clinical practice (Hibbitts and O’Leary, 2018).

Combining nanoparticles with conventional antibiotics has shown significant synergistic effects, enhancing antibacterial efficacy through improved membrane permeability and increased intracellular drug accumulation. For example, AgNP–antibiotic (with ampicillin or vancomycin) combinations demonstrate superior activity against MRSA compared to individual treatments (Gebreyohannes et al., 2019). A combination of AgNPs, visible blue light, and antibiotics (such as azithromycin or clarithromycin) has shown over 8-log10 reduction in MRSA colony-forming units (CFU), offering a potent therapy that can rejuvenate ineffective antibiotics (Akram et al., 2016). Lipid-based nanocarriers, including liposomes and solid lipid nanoparticles (SLNs), further improve therapeutic outcomes by protecting antibiotics from degradation and enabling sustained release (Bazán Henostroza et al., 2022). Notably, engineered systems such as ampicillin-loaded nanosomes, DNase- and penicillin-loaded SLNs, and chitosan-based carriers delivering ursolic acid or chrysin have shown promising activity against pathogenic *S. aureus*. Additionally, advanced nanotherapeutic approaches such as photothermal therapy have shown high specificity; for instance, IgG-conjugated gold nanoparticles selectively target MRSA cells and, upon laser irradiation, induce dose-dependent bacterial cell death, demonstrating their potential as precise and effective antimicrobial agents (Mocan et al., 2016).

Despite these advantages, challenges such as nanoparticle toxicity, biocompatibility, and regulatory limitations remain. Nevertheless, nanomedicine represents a powerful and evolving platform for developing next-generation therapeutics against MRSA infections.

### 9.3 Biomimetic nano-NET-based therapeutic strategies against MRSA

Biomimetic nano-NET strategies represent an advanced therapeutic approach inspired by neutrophil extracellular traps (NETs), which play a crucial role in innate immunity by capturing and eliminating pathogens. These engineered nanoplatforms mimic the natural “trap-and-kill” mechanism of NETs and combine physical bacterial entrapment with chemical or oxidative killing, offering an effective non-antibiotic strategy against multidrug-resistant pathogens such as MRSA (Tram et al., 2023).

Recent developments have demonstrated the potential of NET-inspired nanomaterials. Zhang et al. (2025) designed a biomimetic DNA-based nanomachine with a reticulated structure similar to natural NETs. This nanoplatform exhibited multimodal antibacterial activity by trapping bacteria, disrupting membrane integrity, and inducing leakage of intracellular components, ultimately leading to irreversible bacterial damage. The system also showed high biocompatibility and effectiveness in promoting the healing of infected wounds.

Further advancements have focused on incorporating responsive and regenerative features into nano-NET systems. Xuan et al. (2025) developed photo-responsive amyloid nanoNETs composed of lysozyme-derived nanofibrils. These nanoNETs demonstrated strong antibacterial activity against MRSA while also promoting tissue regeneration. Upon near-infrared stimulation, the system released antimicrobial components and magnesium ions, which enhanced macrophage-mediated healing and reduced inflammation in both murine and porcine infection models.

In addition to nanomaterial-based systems, peptide-engineered NET mimics have also been explored. Li et al. (2026) developed a self-assembling peptide (RFC) that mimics the NET structure and function. This peptide formed nanofibrillar networks capable of entrapping bacteria and disrupting their membranes. It exhibited potent antibacterial activity, significant biofilm inhibition, and high *in vivo* efficacy, including improved survival rates in MRSA-induced sepsis and enhanced wound healing without detectable toxicity.

Complementing these strategies, Xu H. et al. (2025) developed an *ε*-poly-L-lysine-coated copper peroxide nanoplatform (PCPNAs) that integrates biomimetic trapping with chemodynamic therapy. This system releases Cu^2+^ and H₂O₂ under acidic conditions, triggering Fenton-like reactions that generate reactive oxygen species and induce oxidative bacterial death. The positively charged surface enhances interaction with bacterial membranes, enabling efficient bacterial aggregation and biofilm disruption. The nanoplatform achieved significant bacterial reduction and biofilm removal with high biocompatibility in both *in vitro* and in vivo models. Similar ROS-mediated antibacterial effects have also been reported for copper- and iron-based nanocatalysts, while polymer-assisted nanoplatforms improve biofilm penetration and targeting efficiency (Zarepour et al., 2024; Zhai et al., 2025).

Overall, biomimetic nano-NET strategies provide a synergistic approach by combining bacterial entrapment, targeted killing, and immune modulation. These systems offer a promising, biocompatible, and antibiotic-free alternative for the treatment of MRSA infections and other multidrug-resistant bacterial diseases.

### 9.4 Bacteriophage-based therapeutic strategies against MRSA

Phage therapy has emerged as a promising and highly specific approach for the treatment of MRSA. This strategy utilizes bacteriophages viruses that selectively infect bacterial cells to eliminate pathogenic bacteria while sparing the host microbiota (Falagas et al., 2013). Unlike conventional antibiotics, which often disrupt beneficial microbial communities, phage therapy offers targeted antibacterial activity with reduced collateral damage.

A key advantage of bacteriophages is their ability to penetrate and disrupt bacterial biofilms, which are typically resistant to antibiotic treatment and contribute to chronic infections (Bazán Henostroza et al., 2022). Phages attach to bacterial cells, inject their genetic material, and replicate within the host, ultimately leading to bacterial lysis and amplification of the therapeutic effect at the site of infection. Furthermore, phages can be isolated from natural environments such as soil and sewage, screened for activity, and even engineered to enhance their specificity and antibacterial efficiency (Duplessis and Biswas, 2020; Rawat et al., 2023).

Recent studies demonstrate diverse phage-based strategies against MRSA. A bacteriophage cocktail comprising SW21 and SW25 significantly reduced *S. aureus* burden in mammary tissue with efficacy comparable to cephalosporins (Banar et al., 2025). Individual phages such as StAP1 and vB_SauM_VL10 have shown broad lytic activity, with the latter lysing a large proportion of *S. aureus* strains and effectively reducing MRSA biofilms. Similarly, phage WV demonstrated strong bactericidal and antibiofilm effects, which were further enhanced when combined with streptomycin (Jiang et al., 2021). In addition to whole phages, phage-derived proteins such as P128 exhibit potent antimicrobial activity against MRSA (Drilling et al., 2017).

Endolysins represent another promising class of phage-derived therapeutics. Enzymes such as PlySs2, LysK, LysAP45, and LysSte efficiently degrade staphylococcal peptidoglycan and disrupt biofilms at different stages of maturation (Olsen et al., 2018; Sosa et al., 2020; Golosova et al., 2024b) has demonstrated strong antibiofilm activity and efficacy against Gram-positive pathogens (Schuch et al., 2017). Furthermore, engineered lytic proteins including LysH5, CHAP-SH3b, and HydH5-SH3b have shown significant antibiofilm activity (Gutiérrez et al., 2017). Synergistic approaches combining phages or lysins with antimicrobial peptides or antibiotics, such as T7L/T4L with colistin or nisin, Lys11 with R8K, and phage SA46-CTH2 with nisin, have demonstrated enhanced bacterial killing and biofilm eradication (Idelevich et al., 2020; Tyagi et al., 2024). Additionally, combinations such as phiIPLA-RODI with depolymerase Dpo7 further improve antibiofilm activity (Duarte et al., 2024; Table 3).

**Table 3:** Bacteriophage-based therapeutic strategies and phage-derived antimicrobial agents investigated against MRSA, highlighting their antibacterial, antibiofilm, and synergistic therapeutic effects.

Type	Antimicrobial agent	Outcome
Bacteriophage cocktail	SW21–SW25 (vB_SauR_SW21 + SW25)	Significant reduction of *S. aureus* in mammary tissue at 48 h (MOI 10–100); efficacy comparable to cephalosporins (Banar et al., 2025)
Bacteriophage	Phage StAP1 (Herelleviridae)	Lytic phage exhibiting broad-spectrum bactericidal activity against clinical MRSA isolates (Lu et al., 2023)
Bacteriophage	vB_SauM_VL10 (Silviavirus)	Lysed 79.1% of *S. aureus* strains; reduced MRSA biofilm biomass and viability; improved survival in *Galleria mellonella* model (Lerdsittikul et al., 2024)
Bacteriophage	Sb-1	Broad-host-range lytic bacteriophage active against *S. aureus*, including MRSA; effectively reduces biofilms and exhibits synergistic antibacterial activity when combined with antibiotics (Simon et al., 2021)
Bacteriophage	SATA-8505	Lytic bacteriophage with potent activity against clinical MRSA isolates; demonstrates efficient bacterial killing (Pincus et al., 2015)
Phage-derived protein	P128	Exhibits strong antimicrobial activity against *S. aureus*, including MRSA (Drilling et al., 2017)
Endolysins	T7L and T4L	Broad-spectrum antibacterial activity; enhanced efficacy when combined with AMPs (colistin, polymyxin B, nisin; Tyagi et al., 2024)
Endolysin	PlySs2	Effectively reduces biofilms at different maturation stages (Sosa et al., 2020)
Endolysin	LysSte	Hydrolyzes staphylococcal peptidoglycan; demonstrates strong lytic activity (Golosova et al., 2024b)
Endolysin	HY-133	Moderate activity against *S. aureus* on vascular graft-associated mature biofilms (Idelevich et al., 2020)
Bacteriophage	Phage WV	Eliminates most clinical *S. aureus* isolates; shows enhanced antibiofilm and bactericidal effects when combined with streptomycin (Jiang et al., 2021)
Lytic proteins	LysH5, CHAP-SH3b, HydH5-SH3b	LysH5 shows strongest antibiofilm activity; effective screening demonstrated via RTCA technology (Gutiérrez et al., 2017)
Bacteriophage + depolymerase	phiIPLA-RODI + Dpo7	Exhibits synergistic antibiofilm activity against multiple *S. aureus* strains (Duarte et al., 2024)
Endolysin	CF-301 (Exebacase)	Potent antibiofilm activity against staphylococci; effective against other Gram-positive pathogens (Schuch et al., 2017)
Endolysin	LysK	High efficacy against *S. aureus* biofilms; PNAG depolymerases (e.g., DA7) enhance antibiofilm activity (Olsen et al., 2018)
Endolysin	LysAP45	Strong activity against MDR staphylococcal biofilms; thermostable (active after 80 °C exposure; Golosova et al., 2024a)
Endolysin	LysGH15	Exhibits potent bacteriolytic activity against *S. aureus*, including MRSA; effectively disrupts planktonic cells and biofilms, demonstrates synergistic activity with antibiotics (Gu et al., 2011)
AMP + lysin combination	Polymyxin B/colistin/nisin + T7L/T4L	Demonstrates synergistic biofilm eradication (Tyagi et al., 2024)
AMP + lysin combination	R8K + Lys11 (phage ϕ11)	Enhanced bacterial killing due to improved lysin binding to cell wall (Gouveia et al., 2022)
AMP + bacteriophage	Nisin + phage SA46-CTH2	Improved bacterial killing in broth and milk; enhanced biofilm removal (Duc et al., 2020)

Supporting these findings, Samir et al. (2026) reported that phage therapy significantly improved wound healing in MRSA-infected models, with enhanced outcomes when combined with vancomycin. Clinical evidence also highlights its applicability, as Arientová et al. (2026) demonstrated successful systemic phage therapy in an antibiotic-refractory MRSA infection, leading to sustained recovery without relapse.

Despite these promising findings, challenges such as limited understanding of phage biology, manufacturing complexities, and regulatory constraints remain significant barriers to widespread clinical adoption. Strategies such as phage cocktails are being explored to overcome bacterial resistance and improve therapeutic outcomes.

In conclusion, bacteriophage therapy represents a highly targeted and adaptable therapeutic platform against MRSA infections. With continued advancements in phage engineering, formulation, and regulatory frameworks, this approach holds substantial promise as a next-generation alternative or adjunct to conventional antibiotic therapies.

### 9.5 CRISPR-Cas systems in antimicrobial therapy

The CRISPR–Cas system has been emerged as a powerful and versatile platform for both the detection and treatment of infections caused by MRSA. Due to its high sequence specificity and sensitivity, CRISPR-based diagnostics (particularly the Cas12a and Cas13 systems) enable the rapid and accurate detection of pathogen-specific nucleic acids. In some instances, these prove to be more advantageous than traditional PCR-based methods (Gao and Chen, 2025).

CRISPR–Cas systems offer a highly precise antimicrobial approach that selectively eliminates disease-causing bacteria while largely preserving the host’s microbiota. This selectivity is particularly beneficial in eradicating infections caused by drug-resistant MRSA, as it can disrupt specific resistance genes. By targeting factors such as *mecA* or other resistance-associated loci, CRISPR–Cas can effectively restore antibiotic susceptibility without exerting broad-spectrum effects on harmless microbes (Makvandi et al., 2020).

Furthermore, CRISPR-based interventions can eliminate resistance-conferring plasmids, thereby reducing the likelihood of the dissemination and persistence of antimicrobial resistance. Beyond diagnostics, the CRISPR–Cas system has also demonstrated significant potential as a precision antimicrobial agent. Recent studies indicate an inverse relationship between the presence of CRISPR–Cas elements in *S. aureus* and the acquisition of AMR genes, suggesting that CRISPR may play a potential role in limiting horizontal gene transfer (Aman et al., 2020). Therapeutically, CRISPR systems can be engineered to target and eliminate resistance determinants such as *mecA, aacA,* and fluoroquinolone resistance genes (*gyrA/parC*), thereby restoring antibiotic susceptibility.

Furthermore, the CRISPR-mediated cleavage of plasmids carrying resistance genes can effectively “cure” bacteria of these elements, leading to improved treatment outcomes (Fapohunda et al., 2022). Several pre-clinical studies have demonstrated the therapeutic potential of CRISPR-based antimicrobial approaches. Among these, CRISPR-Cas9 has been extensively studied as a programmable antibacterial platform. However, a major challenge lies in the efficient intracellular delivery of Cas9 and single-guide RNA (sgRNA). One strategy to overcome this limitation is to incorporate CRISPR components into phagemids, which can specifically infect bacterial pathogens. For instance, the pDB21*mecA* phagemid has been shown to specifically target *mecA*-positive *S. aureus* clinical strains within a mixed bacterial population. It reduced the proportion of *mecA+ S. aureus* from 50 to 0.4% without affecting non-targeted bacteria (Liu et al., 2023). Similarly, the pDB21aph phagemid, which targets the *aph(3′)* kanamycin resistance gene, proved effective in a mouse skin colonization model. It reduced the proportion of fluorescence-labeled RNKΦ cells from 50 to 11.4%, demonstrating the potential for *in vivo* CRISPR-mediated editing of bacterial populations.

More recently, the CRISPR interference (CRISPRi) system has emerged as a promising tool for targeted gene silencing in *S. aureus*. CRISPRi employs a catalytically inactive Cas9 (dCas9) that retains its ability to bind single-guide RNA (sgRNA) but lacks DNA-cleaving activity (Dong et al., 2017). The resulting dCas9–sgRNA complex binds to specific DNA sequences and blocks RNA polymerase, thereby repressing transcription of one or multiple target genes simultaneously (Jinek et al., 2012; Zhang et al., 2021). Despite its potential for functional genomics and antimicrobial applications, the clinical utility of CRISPRi remains limited by the dose-dependent toxicity of dCas9, commonly referred to as the “bad seed” effect, which requires further optimization (Depardieu and Bikard, 2020).

However, RNA-targeting CRISPR-Cas13a systems have recently emerged as alternative therapeutic options. Unlike Cas9, which cleaves DNA, Cas13a specifically targets and cleaves RNA transcripts, enabling the transient and specific silencing of resistance genes. CRISPR-Cas13a constructs delivered via phagemids have been shown to specifically eliminate bacteria by cleaving the mRNA essential for *mecA* transcription. This ultimately led to bacterial cell death while causing minimal permanent genomic alterations (Shimamori et al., 2024).

These programmable CRISPR platforms expand the therapeutic repertoire against MRSA and provide greater flexibility for targeting diverse resistance mechanisms. Overall, the advances in CRISPR-Cas technology and phage therapy herald a new era of innovative therapeutic strategies aimed at combating AMR while enhancing treatment effectiveness against persistent pathogens like *S. aureus*. Ongoing research is essential to further explore and refine these promising therapeutic strategies.

These programmable CRISPR platforms expand treatment options for MRSA and offer greater flexibility in targeting various resistance mechanisms. Overall, the development of CRISPR-Cas technology and phage therapy points toward a promising direction for future research and innovation in the field of AMR, as well as for the effective treatment of persistent bacterial infections like *S. aureus*. Further studies are needed to better understand and develop these potential therapies.

### 9.6 Probiotic therapeutic agents against MRSA

Probiotics have emerged as a promising biotherapeutic strategy against *S. aureus* and MRSA, owing to their multifaceted antimicrobial and immunomodulatory properties (Le et al., 2021). Unlike conventional antibiotics, probiotics suppress *S. aureus* without exerting strong selective pressure that promotes antimicrobial resistance. Their activity involves multiple mechanisms, including competitive exclusion through nutrient and adhesion site competition, secretion of antimicrobial metabolites such as bacteriocins, organic acids, hydrogen peroxide, and biosurfactants, and enhancement of epithelial barrier function and host immunity. In addition, probiotics stimulate the production of antimicrobial peptides and secretory IgA, while promoting anti-inflammatory cytokines such as IL-10 to limit excessive tissue damage (Zhao et al., 2026).

Probiotics are particularly attractive for MRSA management because the pathogen commonly colonizes the skin, nasal cavity, and gastrointestinal tract, making it difficult to eradicate completely with antibiotics (Barzegari et al., 2020). In addition, MRSA biofilms exhibit high tolerance to antimicrobial agents, contributing to persistent and recurrent infections. Several probiotic microorganisms, including lactic acid bacteria (LAB), *Bifidobacterium* spp., *Bacillus coagulans*, and *Saccharomyces boulardii*, have demonstrated the ability to inhibit MRSA growth and biofilm formation while restoring microbial homeostasis (De Keersmaecker et al., 2006; Tejero-Sariñena et al., 2012; Aw and Fukuda, 2019; Kimelman and Shemesh, 2019).

The therapeutic potential of probiotics has been demonstrated in multiple infection models. *Lactiplantibacillus plantarum* 8P-A3 exhibits potent anti-*S. aureus* activity, significantly reducing skin colonization and preventing wound infections, whereas *Streptococcus salivarius* K12 and M18 display broad-spectrum antagonistic activity against common skin pathogens (Todorov et al., 2019). In chronic wound infections, particularly diabetic ulcers frequently associated with MRSA, topical formulations containing the cell-free supernatant of *Lactiplantibacillus plantarum* MTCC 2621 accelerate wound contraction and tissue regeneration with efficacy comparable to conventional antiseptic therapy in experimental models (Dubey et al., 2023). Furthermore, engineered inactivated *Lacticaseibacillus casei* biofilm coatings represent an innovative approach for preventing implant-associated MRSA infections. These coatings combine strong antibacterial activity mediated by lactic acid and bacteriocin production with enhanced osseointegration through macrophage activation and stimulation of osteogenic signaling, highlighting their potential as multifunctional biomaterials for orthopedic implants (Tan et al., 2020).

Overall, probiotics represent a promising adjunctive approach for preventing and treating MRSA infections by simultaneously targeting bacterial colonization, biofilm formation, and host immune responses. However, further well-designed clinical trials are needed to identify the most effective strains, formulations, and treatment regimens for routine clinical application.

### 9.7 Monoclonal antibodies as therapeutic agents against MRSA

Targeting specific virulence factors of *S. aureus* has emerged as a promising strategy for developing novel therapeutics against bacterial infections. Among these, monoclonal antibodies (mAbs) are engineered proteins that specifically bind and neutralize bacterial virulence determinants, thereby reducing pathogenicity while preserving the host microbiota (Vacca et al., 2022). Unlike conventional antibiotics, mAbs do not exert direct bactericidal activity or impose selective pressure that promotes AMR. Instead, they neutralize toxins such as *α*-toxin, inhibit bacterial adhesion by targeting surface proteins, and enhance host immune responses through opsonization and complement activation (Grooters et al., 2024). Their high specificity minimizes off-target effects, making them attractive therapeutic candidates for MDR pathogens, including MRSA (Falagas et al., 2013; Speziale and Pietrocola, 2021).

Several monoclonal antibodies targeting major *S. aureus* virulence factors have progressed to preclinical and clinical evaluation. Tefibazumab (Aurexis) is a humanized IgG1 antibody directed against clumping factor A (ClfA), a surface adhesin involved in bacterial attachment to fibrinogen and host tissues. Although it significantly reduced mortality in preclinical MRSA bacteremia models, clinical trials demonstrated only modest therapeutic benefit. Another important target is α-toxin (α-hemolysin), a key mediator of tissue damage (McDevitt et al., 1992; McDevitt and Foster, 1995; Hall et al., 2003). AR-301 (KBSA301/tosatoxumab) is a fully human IgG1λ antibody that neutralizes α-toxin and has shown encouraging results as an adjunct to standard antibiotic therapy in patients with severe *S. aureus* pneumonia (François et al., 2018). In contrast, other anti-toxin monoclonal antibodies, including suvratoxumab (MEDI4893) and the dual-antibody cocktail ASN100, demonstrated promising preclinical activity but failed to achieve their primary efficacy endpoints in Phase II clinical trials despite acceptable safety profiles (Yu et al., 2017; François et al., 2018; Magyarics et al., 2019).

Emerging evidence also supports combining monoclonal antibodies with antibiotics. The anti-protein A monoclonal antibody 514G3 promotes opsonophagocytic killing, reduces bacterial burden, and improves survival in experimental models of *S. aureus* bacteremia, highlighting its potential as an adjunctive immunotherapeutic agent (Varshney et al., 2018). Although no monoclonal antibody has yet been approved for the treatment of *S. aureus* infections, advances in antibody engineering, multispecific antibodies, and optimized antibody-antibiotic combination therapies offer promising opportunities for improving the management of severe MRSA infections.

### 9.8 Plant-derived antimicrobial compounds against MRSA

Phytochemicals are gaining increasing attention as promising therapeutic agents against infections caused by MRSA due to their intrinsic antibacterial activity and their ability to potentiate existing antibiotics (Angelini, 2024). These naturally occurring compounds including flavonoids, phenolics, terpenoids, and alkaloids exert their effects through multiple mechanisms such as disruption of bacterial cell membranes, inhibition of virulence factors, modulation of quorum sensing, and interference with biofilm formation (Ghosh et al., 2025). Consequently, plant-derived extracts, essential oils, and purified phytochemicals have emerged as valuable alternatives or adjuncts to conventional antimicrobial therapies (Parimelzanghan et al., 2014; Lavanya et al., 2016; Ragunathan et al., 2019; Mathpal et al., 2025).

Several plant extracts have demonstrated notable anti staphylococcal activity, including methanolic leaf extracts of *Mesua ferrea*, ethanolic bark extracts of *Frangula alnus*, and ethanolic bulb extracts of *Eleutherine americana*. Similarly, essential oils from *Anthemis aciphylla*, *Inula helenium*, and *Turnera subulata* have shown inhibitory effects against *S. aureus*. However, purified phytochemicals such as artonin I, naringenin, and ginsenosides generally exhibit stronger and more consistent antibacterial activity compared to crude extracts (Borges et al., 2015).

Importantly, several phytochemicals enhance antibiotic efficacy through synergistic interactions rather than direct antibacterial action. For instance, anacardic acid and totarol improve the activity of methicillin against MRSA, while berberine exhibits strong synergy with 5′-methoxyhydnocarpin (5′-MHC) by inhibiting efflux pumps and increasing intracellular drug accumulation. Tannins such as tellimagrandin I significantly reduce the minimum inhibitory concentration (MIC) of tetracycline, and compounds like reserpine and capsaicin have been shown to reverse drug resistance, with reserpine notably lowering the MIC of ciprofloxacin against multidrug-resistant strains.

A range of well-characterized phytochemicals further highlights the diversity of mechanisms involved. Alkaloids such as berberine (from *Berberis vulgaris* and *Coptis chinensis*) inhibit DNA gyrase and topoisomerase IV while blocking efflux pumps (Aksoy et al., 2020). Flavonoids such as epigallocatechin gallate (EGCG) target ABC transporters and resistance-associated proteins (e.g., MecA), reduce virulence factors like α-hemolysin, and inhibit biofilm formation (Knidel et al., 2021). Polyphenols such as carnosol (*Rosmarinus officinalis*) and curcumin (*Curcuma longa*) interfere with bacterial metabolism, quorum sensing, and biofilm development, with curcumin also inducing ROS-mediated killing effects that can be further enhanced through nanoparticle-based delivery systems (Chen et al., 2004; Shen et al., 2020; Qin et al., 2023; Zhou et al., 2025).

Monoterpenoid phenols such as thymol and carvacrol disrupt bacterial membrane integrity and inhibit quorum sensing, while thymol derivatives (e.g., TPP–thymol conjugates) have demonstrated rapid killing of MRSA persister cells in both *in vitro* and *in vivo* models (Shahi et al., 2024; Tang et al., 2024). Similarly, trans-cinnamaldehyde exhibits strong antibacterial and antivirulence activity by inhibiting growth and biofilm formation. Other compounds, including baicalin and tanshinone IIA, inhibit biofilm formation and bacterial adhesion, with improved efficacy when delivered via nanocarriers (Abdel-Raheem et al., 2023).

Additional phytochemicals such as piperine (*Piper nigrum*) significantly inhibit MRSA biofilm formation at low concentrations (Das et al., 2021) while ent-kaurane diterpenoids (*Siegesbeckia orientalis*) disrupt bacterial cellular integrity (Yang et al., 2025). Anthocyanins from *Lycium ruthenicum* and tannins from *Penthorum chinense* have been shown to damage biofilm structure, reduce extracellular polymeric substances, and regulate key virulence-associated genes such as *icaA*, *sarA*, *cidA*, *sigB*, and *agrA* (Dong et al., 2022). Moreover, compounds like β-citronellol and geraniol (*Pelargonium graveolens*) exhibit anti-adhesive properties and interact with regulatory proteins such as SarA, further inhibiting virulence expression (Elghali et al., 2024; Table 4).

**Table 4:** Plant-derived compounds and phytochemicals investigated against MRSA, highlighting their antibacterial, antibiofilm, antivirulence, and resistance-modulating activities.

Type	Antimicrobial agent	Outcome
Alkaloid	Berberine (*Berberis vulgaris*, *Coptis chinensis*)	Intercalates bacterial DNA, inhibits cell division, suppresses NorA-mediated efflux pumps, and potentiates conventional antibiotics (Aksoy et al., 2020)
Flavonoid	Epigallocatechin gallate (EGCG) (*Camellia sinensis*)	Binds to ABC transporters and MecA protein; downregulates resistance-associated proteins; reduces virulence (α-hemolysin) and inhibits biofilm formation (Knidel et al., 2021)
Phenolic diterpene	Carnosol (*Rosmarinus officinalis*)	Disrupts bacterial metabolism, inhibits quorum sensing and biofilm formation, and attenuates MRSA virulence (Shen et al., 2020)
Monoterpenoid phenol derivative	Thymol derivatives (TPP–thymol conjugates)	Rapidly eradicate MRSA persister cells by disrupting membrane integrity; exhibit potent antibacterial efficacy *in vitro* and *in vivo* (Tang et al., 2024)
Monoterpenoid	Carvacrol	Disrupts cell membrane integrity, inhibits quorum sensing and virulence, effective against MDR-MRSA (Abdel-Raheem et al., 2023)
Phenylpropanoid	Trans-cinnamaldehyde	Exhibits strong antibacterial and antivirulence activity, inhibits MRSA growth and biofilm formation (Abdel-Raheem et al., 2023)
Polyphenols (tannins)	*Terminalia chebula*, *Camellia sinensis*	Inhibit biofilm formation, promote wound healing, and modulate host immune responses by enhancing macrophage activation (Qin et al., 2023)
Polyphenol	Curcumin (*Curcuma longa*)	Inhibits quorum sensing and biofilm formation, induces ROS-mediated bacterial killing, disrupts membrane integrity, and exhibits enhanced efficacy through nanoparticle delivery (Zhou et al., 2025)
Flavonoid	Baicalin	Exhibits antibiofilm activity, interferes with quorum sensing and reduces virulence expression (Zhou et al., 2025)
Diterpenoid	Tanshinone IIA	Inhibits bacterial adhesion and biofilm formation and suppresses virulence, nanocarrier formulations improve antibacterial efficacy (Zhou et al., 2025)
Monoterpenoid phenol	Thymol	Exhibits antibacterial activity, derivatives show improved potency against MRSA with low MIC values (Shahi et al., 2024)
Alkaloid	Piperine (*Piper nigrum*)	Inhibits MRSA biofilm formation and enhances intracellular antibiotic accumulation through efflux pump inhibition (Das et al., 2021)
Diterpenoid	ent-Kaurane diterpenoids (*Siegesbeckia orientalis*)	Strong anti-MRSA activity, disrupt bacterial integrity and cellular processes (Yang et al., 2025)
Anthocyanin	*Lycium ruthenicum*	Inhibit initial biofilm formation and disrupt the biofilm structure (Dong et al., 2022)
Tannin	TPCP (*Penthorum chinense*)	Eradicates established biofilms, reduces extracellular polysaccharides and eDNA production, and downregulates *icaA, sarA, cidA, sigB*, and *agrA* expression (Qin et al., 2023)
Monoterpenoid alcohols	β-Citronellol and geraniol (*Pelargonium graveolens*)	Reduce bacterial adhesion and inhibit SarA-mediated biofilm formation through interaction with the SarA regulator (Elghali et al., 2024)
Stilbenoid polyphenol	Resveratrol (*Vitis vinifera*)	Inhibits biofilm formation, suppresses quorum sensing and virulence gene expression, reduces bacterial adhesion, and potentiates antibiotic activity against MRSA (Cui et al., 2024)
Pentacyclic triterpenoids	Ursolic acid/Oleanolic acid	Potentiate β-lactam antibiotics by interfering with PBP2a-mediated resistance and restoring β-lactam susceptibility in MRSA.(Catteau et al., 2017)
Flavonoid	EGCG (*Camellia sinensis*)	Inhibits β-lactamase activity, suppresses PBP2a function, and enhances the efficacy of β-lactam antibiotics against MRSA (Zhao et al., 2002)
Polyketide metabolite	*Aranorosin (Gymnascella aurantiaca)*	Inhibits the bifunctional aminoglycoside-modifying enzyme (Labby and Garneau-Tsodikova, 2013)
Organosulfur compound	Allicin (*Allium sativum*)	Inhibits thiol-containing enzymes, disrupts bacterial metabolism, and exhibits synergistic antibacterial activity with β-lactams and glycopeptides (Hoseini Alfatemi et al., 2014)
Phenolic acid	Caffeic acid	Suppresses biofilm formation, inhibits bacterial adhesion, and enhances the antibacterial efficacy of conventional antibiotics (Kępa et al., 2018)
Phenolic acid	Gallic acid	Disrupts bacterial membrane integrity, inhibits biofilm formation, and exhibits synergistic activity with antibiotics (Sang et al., 2024)
Anthraquinone	Emodin	Inhibits MRSA growth, suppresses biofilm formation, and virulence gene expression (Chu et al., 2025)

Beyond their direct antibacterial effects, many phytochemicals exhibit immunomodulatory properties that can aid in improving infection control. These natural compounds can influence protein–protein interactions involved in immune signaling, inflammation, and host defense mechanisms. Extracts from *Aloe vera* and *Bacillus ambrosioides* are known to enhance the phagocytic activity of macrophages (Spellberg et al., 2015), while *Panax ginseng* saponins and *Emblica officinalis* extracts stimulate T- and B-cell responses, thereby strengthening both innate and adaptive immunity (Kang and Min, 2012). These host-directed effects can complement antibacterial activity and improve pathogen elimination, particularly in chronic or recurrent infections.

Despite promising preclinical findings, the clinical application of phytochemicals remains limited. Most evidence stems from *in vitro* experiments or animal studies, whereas robust data regarding pharmacokinetics, pharmacodynamics, toxicity, and efficacy in humans are scarce. Many phytochemicals suffer from poor aqueous solubility, low oral bioavailability, rapid metabolism, and batch-to-batch variability arising from differences in plant sources, extraction processes, and phytochemical composition. Furthermore, there is a lack of standardized formulations, optimal dosing regimens, and large-scale clinical trials, all of which constrain regulatory approval and clinical use.

Overall, phytochemicals represent a diverse and multifunctional class of anti-MRSA agents. Their ability to target multiple pathways including membrane integrity, enzymatic activity, quorum sensing, and biofilm formation/combined with their synergistic interactions with antibiotics, positions them as promising candidates for the development of next-generation antimicrobial strategies.

## 10 Clinical translation challenges and future perspective

In recent years, significant progress has been made in the field of biomedical science, leading to the emergence of novel approaches to overcome the growing problem of AMR. These approaches include innovative strategies such as CRISPR-Cas systems, monoclonal antibodies, bacteriophage therapy, nanomedicine-based drug delivery, plant-derived antimicrobial compounds, and drug repurposing. While these methods hold promise, their successful application in clinical settings faces numerous challenges. Although many of these approaches have proven highly successful in laboratory and animal studies, very few have advanced to the final stages of clinical trials or clinical practice. This discrepancy creates a critical clinical translation gap, as promising experimental results often fail to translate into therapies that are standardized, safe, scalable, and cost-effective.

Bacteriophages therapy has been shown promising efficacy and safety against drug-resistant *S. aureus* infections, however their use in clinical practice is still limited. Key challenges include regulatory variations, lack of standardisation regarding the phage production, purification and potency testing as well as the need for strain specificity in phage selection (Zhao et al., 2026). Furthermore, critical issues such as the emergence of phage resistance, patient selection for phage therapy, and the long-term impact of phages on the host microbiome require further in-depth research (Kolenda et al., 2024). The future efforts should focus on improving the knowledge of the phage-host interactions, such as developing phage libraries and optimizing the phage cocktail composition to avoid bacterial resistance mechanisms. Besides, the research of advanced phage engineering techniques, such as CRISPR-Cas technology, may greatly enhance the efficacy and specificity of phage therapeutics.

Likewise, probiotics have demonstrated considerable potential as an adjunctive treatment for *S. aureus*, although their clinical application remains limited due to the absence of standardized treatment guidelines and a lack of consistency in clinical evidence. Treatment outcomes across various studies differ based on factors such as strain variations, metabolite production, host variants, treatment modalities, and experimental models (Mazurak et al., 2015). Additionally, issues regarding quality control, strain identification, long-term safety, and intestinal colonization must be addressed before probiotics can be routinely adopted in clinical practice (da Silva et al., 2024; Chen et al., 2025). CRISPR-based antimicrobial strategies are highly precise and programmable; there are several biological and technical challenges that hinder their clinical translation to eliminate MRSA. Efficient delivery of CRISPR components into biofilm-associated bacteria remains a major obstacle because the extracellular polymeric matrix limits penetration and reduces delivery efficiency. While delivery methods like viral vectors, lipid nanoparticles, and polymeric carriers have shown promise, there is a need for further optimization to enhance their efficiency and stability (Lee et al., 2025; Seijas et al., 2025). However, there are concerns that need to be addressed before clinical application, such as off-target genome editing, chromosomal rearrangements, and host immune responses (Hunt et al., 2023; Lotfi et al., 2025; Xu et al., 2026). Moving forward, there will be a need for further studies to enhance delivery platforms and create highly specific, self-targeting CRISPR systems to help fight MRSA infections associated with biofilms. Despite differences in their mechanisms of action, bacteriophages, probiotics, and CRISPR-based therapeutics face several common translational barriers. These include the lack of standard manufacturing and quality control procedures, targeted delivery challenges, limited large scale clinical evidence and regulatory uncertainty. Addressing these common challenges will be crucial to making these technologies a viable treatment option. Nanoparticle-based therapies have demonstrated promising antibacterial and antibiofilm activity; however, their clinical translation is limited by unresolved issues related to long-term toxicity, biodistribution, and biocompatibility (Smith and Gambhir, 2017). Furthermore, large-scale, reproducible, and cost-effective manufacturing remains a challenge, while existing regulatory frameworks are not fully equipped to evaluate nanomaterial-based therapeutic methods (Makabenta et al., 2021) However, despite these limitations, the advent of nanotechnology allows for their incorporation into wound dressings, catheters, and antibiotic combination therapies, thereby improving treatment outcomes and reducing the development of antibiotic resistance (Gupta et al., 2019; Karnwal et al., 2023; Xu et al., 2023).

AMPs exhibit potent antibacterial and antibiofilm properties; however, their therapeutic application is limited by low stability, rapid proteolytic degradation, short *in vivo* half-life, and potential cytotoxicity (Lai et al., 2022; Di Donato et al., 2025). Although chemical modifications, peptide engineering, and nanocarrier-based delivery systems have substantially improved their stability and pharmacokinetic properties, these approaches often increase manufacturing complexity and production costs (Ouyang et al., 2025). Using AMPs alongside traditional antibiotics is a potential way to enhance the effectiveness of antibiotics and delay the development of resistance. Future research should prioritize improving pharmacokinetics, confirming efficacy in clinically relevant models, and integrating computational peptide design with advanced delivery technologies (Kantroo et al., 2025; Ma et al., 2025; Matei and Visan, 2025; Shang et al., 2025).

Monoclonal antibodies (mAbs) offer a targeted therapeutic approach against *S. aureus* virulence factors. Despite their promising potential, their clinical translation remains limited by antigenic variability, Protein A-mediated immune evasion, biofilm-associated protection, and intracellular persistence. Furthermore, several clinical trials have also reported inconsistent efficacy of mAbs, suggesting that targeting a single virulence factor may be insufficient. Future strategies should focus on engineered multispecific antibodies and their combination with conventional antibiotics to improve therapeutic outcomes (Speziale et al., 2018; Raafat et al., 2019).

Overall, despite significant progress in these novel and potent therapeutic approaches, their adoption in clinical practice has been slow. Major barriers to implementation include a lack of clinical evidence, the absence of standardized manufacturing and regulatory guidelines, delivery challenges, safety concerns, and issues related to biofilm-associated infections. Furthermore, despite the wide range of compounds available, inconsistencies in clinical trial designs, the lack of validated markers to measure therapeutic efficacy, and limited data on long-term safety and effectiveness pose challenges for regulatory approval and widespread clinical use. Therefore, future efforts should focus on multicenter randomized clinical trials, standardized assessment methods, large-scale production techniques, and interdisciplinary research and development involving clinicians, microbiologists, bioengineers, and regulatory authorities. Combination therapies alongside precision medicine and AI-driven therapy design, could accelerate the translation of these potent strategies into effective clinical treatments for multidrug-resistant *S. aureus* infections.

## 11 Conclusion

MRSA continues to evolve as a major MDR pathogen, with the ability to develop resistance against both conventional and newly introduced antibiotics. The increasing prevalence of biofilm-associated infections, virulence adaptation, and diverse resistance mechanisms has made the treatment of MRSA increasingly difficult and has highlighted the limitations of currently available therapies. The evidence presented in this review underscores the potential of several emerging therapeutic strategies to overcome the limitation of current therapeutic options. Emerging approaches such as antimicrobial peptides, nanomedicine-based drug delivery systems, bacteriophage therapy, CRISPR-Cas technologies, biomimetic nano-NETs, probiotics, monoclonal antibodies and plant-derived compounds have shown significant potential in improving antibacterial activity, disrupting biofilms, enhancing targeted drug delivery, and reducing the development of resistance. Several of these strategies also exhibit synergistic effects when combined with existing antibiotics, thereby improving therapeutic efficacy against resistant strains. Although several challenges related to safety, clinical validation, and large-scale application still remain, these emerging therapeutics provide promising alternatives for future MRSA management. Continued research and the development of novel antimicrobial strategies will be essential to combat the ongoing and future threat of antibiotic-resistant MRSA infections.
